# A detailed investigation of rare earth lanthanum substitution effects on the structural, morphological, vibrational, optical, dielectric and magnetic properties of Co-Zn spinel ferrites

**DOI:** 10.3389/fchem.2024.1433004

**Published:** 2024-08-30

**Authors:** Anam Hameed, Ali Asghar, Saqib Shabbir, Ishfaq Ahmed, Ayesha Khan Tareen, Karim Khan, Gulzar Hussain, Majed Yousef Awaji, Hafeez Anwar

**Affiliations:** ^1^ Department of Physics, University of Agriculture Faisalabad, Faisalabad, Pakistan; ^2^ Shenzhen University, Shenzhen, China; ^3^ School of Materials Science and Engineering, Harbin Institute of Technology, Harbin, China; ^4^ Dongguan University of Technology, Dongguan, Saudi Arabia; ^5^ Department of Physics, Faculty of Science, Jazan University, Jazan, Saudi Arabia; ^6^ Institute Jean Lamour, University of Lorraine, Nancy, France

**Keywords:** sol-gel auto combustion, rare earth spinel ferrites, optical bandgap, dielectric materials, conductivity, magnetization

## Abstract

In this work, Co_0.5_Zn_0.5_La_x_Fe_2-x_O_4_ (0.00 ≤ x ≤ 0.10) spinel ferrites were synthesized using the sol-gel auto-combustion method. X-ray diffraction (XRD) analysis and Rietveld refinement confirmed the presence of a cubic spinel structure. The crystallite size was estimated to be between 17.5 nm and 26.5 nm using Scherrer’s method and 31.27 nm–54.52 nm using the Williamson–Hall (W-H) method. Lattice constants determined from XRD and Rietveld refinement ranged from (8.440 to 8.433 Å and 8.442 to 8.431 Å), respectively. Scanning electron microscopy (SEM) revealed a non-uniform distribution of morphology with a decrease in particle size. The bandgap values decreased from 2.0 eV to 1.68 eV with increasing rare earth (La^3+^) doping concentration. Fourier-transform infrared (FT-IR) spectroscopy confirmed the presence of functional groups and M-O vibrations. The dielectric constant and dielectric loss exhibited similar behavior across all samples. The maximum tan δ value obtained at lower frequencies. Regarding magnetic behavior, there was a decrease in magnetization from 55.84 emu/g to 22.08 emu/g and an increase in coercivity from 25.63 Oe to 33.88 Oe with higher doping concentrations. Based on these results, these materials exhibit promising properties for applications in microwave and energy storage devices.

## 1 Introduction

Nanocrystalline spinel ferrites have garnered significant attention in the scientific community due to their distinctive structural, optical, dielectric and magnetic characteristics. These ferrites are found in applications such as sensors, supercapacitors, magnetic substances, magneto-thermal energy storage systems, magnetic drug delivery and hypothermia therapy specifically for cancer patients (Nikam et al., 2014; Kiani et al., 2022). Among spinel ferrites, cobalt ferrite has garnered considerable research interest for medical and secondary storage device applications (Asghar et al., 2022; Mısırlıoğlu et al., 2022). With a moderate saturation magnetization, cobalt ferrite exhibits notable coercivity. The introduction of non-magnetic Zn^2+^ ions allowed tailoring of ferrite properties for various purposes (Andhare et al., 2020). Dielectric and magnetic properties of ferrites can be enhanced by incorporating trivalent rare-earth ions (Li et al., 2021). Even a modest amount of RE substitution can lead to improved dielectric and magnetic attributes in ferrites (Tanbir et al., 2020; Suo et al., 2021). For instance, replacing nanocrystalline CoFe_2_O_4_ with La, Gd, Sm, or Nd reduces the dielectric constant (ɛʹ) with increasing RE ion concentration (Nikumbh et al., 2014). Substituting larger radius RE ions effectively has altered the ferrite’s Curie temperature. Nd^3+^ ion substitution enhanced the coercivity of cobalt ferrite (Muskan et al., 2024; Reddy et al., 2022). This substitution reduced the ferrite’s dielectric loss and enhanced usability. Ni ferrite’s structural and magnetic traits improved significantly with rare-earth substitution (Lumina et al., 2018).

Within the lanthanides group, lanthanum (La^3+^) stands out due to its significant atomic size, despite being non-magnetic (Mariño-Castellanos et al., 2021). The advantage of La for substitution in Co_0.5_Zn_0.5_La_x_Fe_2-x_O_4_ spinel ferrites is due to its relatively suitable ionic radius, chemical stability, and impact on magnetic interactions. La also has the potential to modify the electronic structure and enhance dielectric and microwave absorption properties. These factors collectively make La an ideal choice for advancing the understanding and application of rare earth-substituted ferrites. The substitution of RE with Fe^3+^ cations introduced strain into the lattice ferrites, thereby altering the crystal size. Given its singular +3 valence state, La^3+^ readily occupies octahedral [B] lattice sites within the ferrite structure, consequently modifying magneto-structural characteristics. The impact of the structure of La^3+^ ions on the magneto-structural, dielectric, and magnetic properties has been studied deeply. In La-substituted Cu-Zn ferrites, it has been observed that the dielectric constant and loss tangent increase as temperature rises (Patil et al., 2023). Furthermore, the addition of La^3+^ to Co ferrite results in alterations to the super-exchange interactions between Fe^3+^ and Fe^2+^ ions (Haque et al., 2017). The replacement of larger La^3+^ cations in Mn-Cr ferrites leads to lattice deformation, which in turn influences magnetic properties and structural characteristics (Abdellatif et al., 2018). In the case of Ni-Co ferrites, the substitution of Sm^3+^ ions diminish their magnetic features, rendering them suitable for use in recording and memory devices (Kokare et al., 2019). Lastly, the introduction of La^3+^ ions into Co ferrites results in a reduction of its magnetic attributes, rendering it superparamagnetic in nature (Gaba et al., 2018).

This research investigates the effect of rare earth element La^3+^ on the morphological, optical, dielectric and magnetic properties of Co_0.5_Zn_0.5_La_x_Fe_2-x_O_4_ (x = 0.00, 0.025, 0.050, 0.075, 0.10), synthesized using the auto-combustion technique. The amount of Zn concentration is fixed at 0.5 for several reasons such as maintaining a constant Zn level allowed us to systematically explore the effects of La substitution on Co-Zn ferrite. This approach ensured that any observed changes in properties were directly attributable to La substitution, yielding more consistent and comparable results. The substitution of La results in a decrease in the band gap of the prepared samples from 2.0 to 1.68 eV, making these materials promising candidates for photocatalysis. Additionally, the replacement of Fe^3+^ with non-magnetic rare earth cations weakens Fe^3+^-Fe^3+^ interactions, affecting not only the magnetic properties but also inducing changes in the microstructures. The peak values of the loss tangent (tanδ) indicate an enhancement in both microwave absorption and dielectric properties (Ren and Xu, 2014).

## 2 Materials and method

### 2.1 Reagents

The synthesis of Co_0.5_Zn_0.5_La_x_Fe_2-x_O_4_ (x = 0.00, 0.025, 0.050, 0.075, 0.10) involves the utilization of the following components in their respective forms: ferric nitrate non-ahydrate [Fe(NO_3_)_3_·9H_2_O], cobalt nitrate hexahydrate [Co(NO_3_)_2_·6H_2_O], ammonium hydroxide (NH_4_OH), zinc nitrate hexahydrate [Zn(NO_3_)_2_·6H_2_O], lanthanum nitrate hexahydrate [La(NO_3_)_3_·6H_2_O] and citric acid (C_6_H_8_O_7_). The solutions are prepared using deionized water. All compounds are directly used without any additional processing.

### 2.2 Experimental section

**FIGURE 1:**
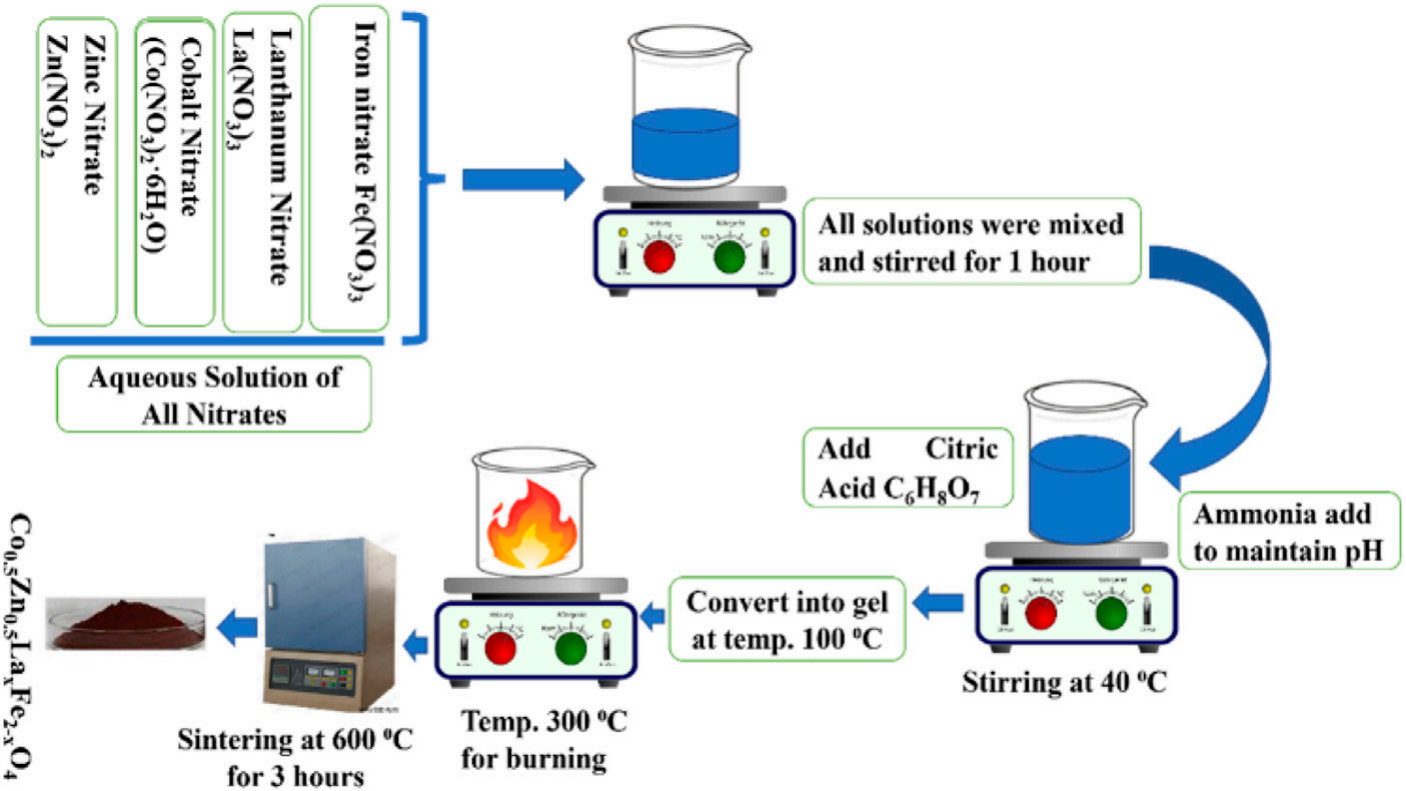
Preparation of Lanthanum substitution cobalt zinc spinel ferrites.

The Co_0.5_Zn_0.5_La_x_Fe_2-x_O_4_ (x = 0.00, 0.025, 0.050, 0.075, 0.10) spinel ferrites were prepared using the sol-gel auto combustion technique, as illustrated in Figure 1. The sol-gel auto-combustion method is distinguished by its versatility and efficiency in synthesizing spinel ferrites, allowing for precise control over composition, morphology, and uniformity. The synthesis involved the use of La(NO_3_)_3_·6H_2_O, Zn(NO_3_)_2_·6H_2_O, Fe(NO_3_)_3_·9H_2_O and Co(NO_3_)_2_·6H_2_O based on stoichiometric calculations. These salts were mixed in distilled water (DI) in accordance with stoichiometric ratios and then subjected to agitation to achieve a homogeneous solution. The prepared solution was placed on a magnetic stirrer and citric acid was added to the solution to act as a fuel agent alongside the nitrates.

The solution’s pH was sustained at 7 through the gradual addition of ammonia solution. Subsequently, the temperature was raised from 100°C to 110°C until the sample transformed into a gel-like state. Stirring was sustained until combustion took place. A comparison of the synthesis conditions and parameters for the present study with those from prior literature is presented in Table 1. The resulting ash was obtained and the prepared samples were sintered at 750°C for 4 h in a furnace to remove volatile substances like moisture and other undesirable elements. The sintered powders were then subjected to analysis using various procedures, including X-ray diffraction, scanning electron microscopy, energy-dispersive X-ray analysis, Fourier-transform infrared spectroscopy, UV-visible spectroscopy, LCR measurements and vibrating sample magnetometry to characterize their properties.

**TABLE 1:** Comparison of synthesis conditions/parameters for current work with previous literature.

Material	Method of preparation	Dissolving media	Sintering temperature (°C)	Time of sintering (h)	Ref
Co_0.5_Zn_0.5_La_x_Fe_2-x_O_4_	Sol-gel	Distilled water	—	—	Gilani et al. (2020)
Zn_0.5_Co_0.5_La_x_Fe_2-x_O_4_	Co-precipitation	De-ionized water	750	—	Aslam et al. (2021)
Co_0.1_Zn_0.9_La_x_Fe_2-x_O_4_	Citrate gel auto-combustion	Double-distilled water	500	4	Sumalatha et al. (2022)
La-substituted Zn–Co–La ferrite	sol-gel	—	1,400	5	Dippong and Mereu (2024)
Co_0.5_Zn_0.5_La_x_Fe_2-x_O_4_	sol-gel auto combustion	Distilled water	600	4	Kulkarni and Rathod (2016)
Co_0.7_Zn_0.3_La_x_Fe_2-2x_O_4_	Sol-gel route	Deionized water	400	—	Mugutkar et al. (2022)
Co_0.65_Zn_0.35_La_x_Fe_2-x_O_4_	Flash method	—	800	—	Altarawneh et al. (2023)
Co_0.7_Zn_0.3_La_x_Fe_2-x_O_4_	Sol gel auto ignition route	Aqueous solutions	450	—	Mugutkar et al. (2020)
Co_0.5_Zn_0.5_La_x_Fe_2-x_O_4_	Auto-combustion	Distilled water	750	1	Current work

### 2.3 Characterization

The X-ray diffractometer (XRD), which operated with a precise wavelength (λ) of 0.154 nm and covered a 2θ range spanning from 20° to 80°, was utilized for capturing diffraction patterns. The scanning electron microscope played a pivotal role in elucidating external appearance and crystallographic structure. FTIR spectroscopy, specifically using a Perkin instrument, was employed to validate the absorption bands (ν_1_, ν_2_) within the wavenumber range of 4,000 to 400 cm⁻^1^. The samples’ absorption spectra were documented employing the UV-Vis spectrophotometer PG (Model T-80). Dielectric measurements were performed at room temperature (RT) using the IM-3536 series LCR Meter and Impedance Analyzer. Lastly, a VSM investigation was conducted to ascertain the M-H hysteresis loops of Co_0.5_Zn_0.5_La_x_Fe_2-x_O_4_.

## 3 Results and discussion

### 3.1 Structural analysis

**FIGURE 2:**
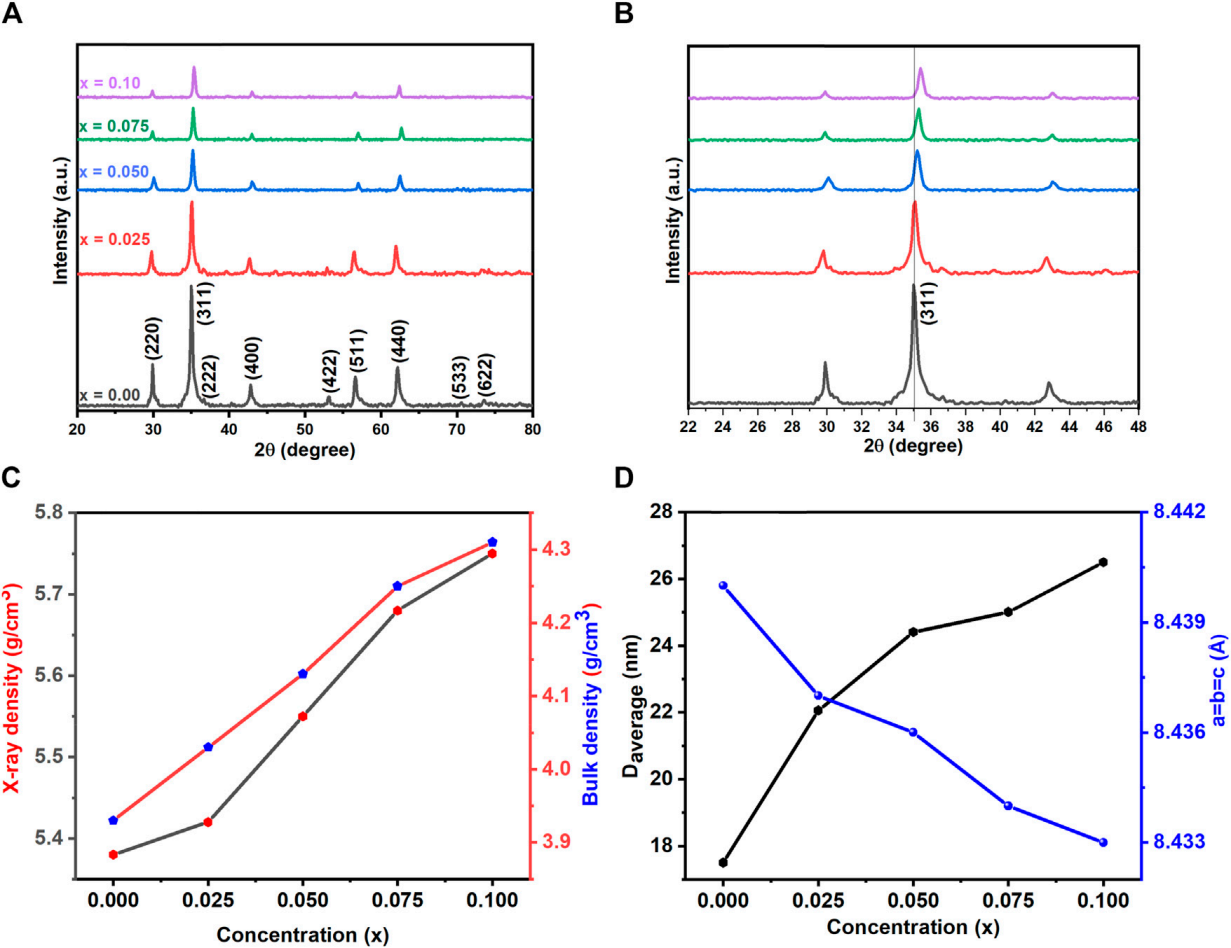
**(A)** XRD pattern **(B)** enlarged view of XRD **(C)** effect of La concentration on D_average_, Lattice constant (a = b = c) **(D)** ρ_x-ray_ and bulk density of Co_0.5_Zn_0.5_La_x_Fe_2-x_O_4_ (x = 0.00, 0.025, 0.050, 0.075, 0.10) spinel ferrites.

**FIGURE 3:**
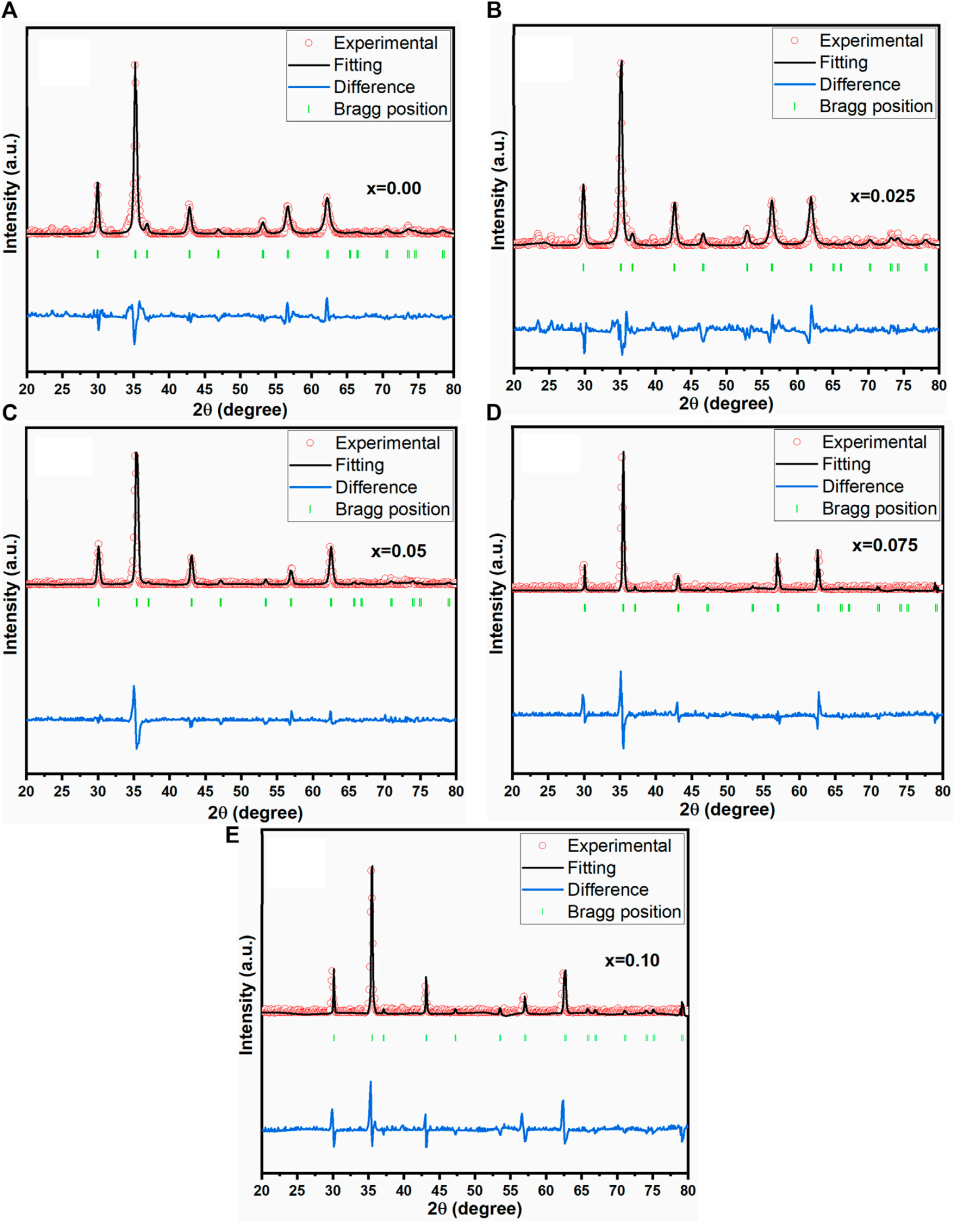
Rietveld refined pattern of the Co_0.5_Zn_0.5_La_x_Fe_2-x_O_4_ **(A)** x = 0.00 **(B)** x = 0.025 **(C)** x = 0.05 **(D)** x = 0.075 **(E)** x = 0.10.

Figure 2A displays the X-ray diffraction pattern of as-prepared spinel ferrites synthesized through the auto-combustion process. The discernible peaks (220), (110), (311), (400), (422), (511), (440), (533) and (622) significantly confirmed the existence of a single-phase cubic structure, corresponding to the JCPDS file no: 22-1086 (Guo et al., 2022). In Figure 2B, the peak (311) exhibited a shift towards a higher angle. It is worth noting that the preparation of this material is reproducible, as it prepared many times for other investigations. To further investigate crystal structures, Rietveld refinement plots were generated for synthesized spinel ferrites, refer to Figures 3A–E. These findings align with prior data, providing further assurance of the nanocomposites’ composition and crystal structures. For the Rietveld refinement process, the FULLPROF software was utilized, and the Thompson-Cox-Hastings pseudo-Voigt function was employed to model the Bragg peaks (Abbas et al., 2023; Ahmed et al., 2023; Shabbir et al., 2024). A polynomial function with six coefficients was utilized for background calculation. The parameters used for fitting, including weighted profile R-factor (R_wp_), unweighted profile R-factor (R_exp_), GoF and chi-square (χ^2^), have been recorded in Table 2.

**TABLE 2:** Data was obtained through Rietveld refinement for prepared samples.

Concentration	R_wp_ (%)	R_exp_ (%)	χ^2^	GoF	a = b = c (Å)	Volume (Å)^3^
0.00	25.6	10.5	2.43	15.2	8.442	601.639
0.025	20.4	8.2	2.48	13.7	8.438	600.784
0.05	23.3	9.7	2.40	17.5	8.436	600.357
0.075	24.1	9.9	2.43	19.1	8.435	600.143
0.10	22.2	8.6	2.58	16.8	8.431	599.290

The weighted profile R-factor (R_wp_) in the provided data ranges from 20.4 to 25.6, indicating a trend of improvement in the fit between observed and calculated diffraction patterns as the concentration of Co-Zn spinel ferrites increases. Correspondingly, the expected R-factor (R_exp_) values decrease from 10.5 to 8.2, supporting the notion of enhanced fit with higher concentrations. The chi-square (χ^2^) values, ranging from 1.23 to 1.58, are relatively close to 1, suggesting good agreement between observed and calculated data. Notably, the lowest R_wp_, R_exp_, and χ^2^ values are observed at a concentration of 0.025, indicating that the crystal structure refinement at this specific concentration yields the best agreement with experimental data. This concentration appears to be optimal for achieving an improved fit in the context of the investigated Co-Zn spinel ferrites.

Notably, at x = 0.05, a relatively low-intensity peak (110) appears around 33°, indicating the presence of the α-Fe_2_O_3_ phase. This might be associated with the dissimilarity in ionic radius between the substituted rare earth cations and the original Fe^3+^. The XRD graphs affirmed the development of a solitary geometric cubic pattern for lanthanum-substituted cobalt spinel ferrites, without any secondary phase. Scherrer equation (mentioned as Equation 1) was utilized to determine the crystallite size (D) of the synthesized samples (Ahmed et al., 2022; Hussain et al., 2022):

$$D=\frac{K\lambda }{\beta \mathrm{Cos}\theta }$$ (1)

where k, λ, β and θ represent shape factor, wavelength 1.54 (Å), peak broadening, and diffraction angle, respectively. The values of crystallite size increase with increasing the lanthanum substitution as shown in Figure 2C. With increasing RE (La) doping concentration, the crystallite size generally increases, signifying potential effects on magnetic and electrical properties, and suggesting alterations in synthesis or annealing conditions. The following Equation 2 is used to measure micro-strain:

β=4×ε×⁡tanθ (2)

The observed peak broadening in Williamson–Hall analysis results from the summation of Equations 1, 2, as expressed by the following formulation (Equation 3):

$$\beta \times \cos\theta =\left(0.9\lambda /D\right)+\left(4\times \text{Strain}\times \sin\theta \right)$$ (3)

**FIGURE 4:**
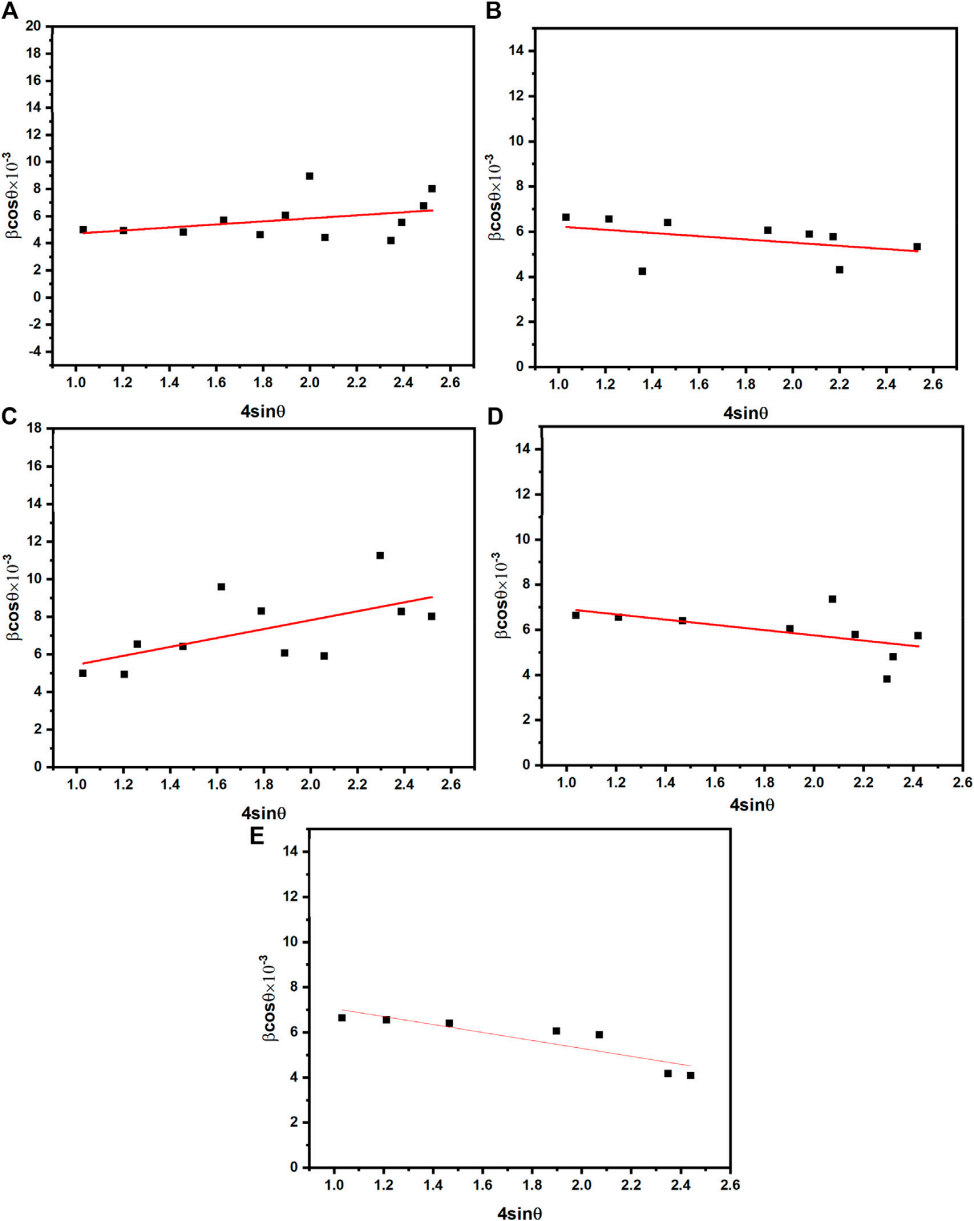
**(A–E)** The W-H plots of Co_0.5_Zn_0.5_La_x_ Fe_2-x_O_4_ (x = 0.00, 0.025, 0.05, 0.075, 0.10).

The straight line represents the slope value obtained from the relationship between β × cosθ and 4sinθ. Micro-strain is then calculated based on the slope values Figures 4A–E. The lattice constant of prepared samples was calculated by the following Equation 4:

$$\frac{1}{d^{2}}=\frac{h^{2}}{a^{2}}+\frac{k^{2}}{b^{2}}+\frac{l^{2}}{c^{2}}$$ (4)

In the equation, where h, k, l, and d denote the Miller indices and d-spacing, respectively. The lattice constant’s trend is observed to decrease with increasing lanthanum substitution, as depicted in Figure 2C.

This phenomenon can be ascribed to the existence of larger RE cations, which have an ionic radius of (1.061 Å), in contrast to the smaller ionic radius of Fe^3+^ ions (0.67 Å) (Mahmood and Maqsood, 2021). The unit cell volume (V_cell_) of the synthesized samples was calculated using the following Equation 5:

$$V=a^{3}$$ (5)

An observable trend emerged as lanthanum substitution increased, resulting in a decrease in the unit cell volume (V_cell_) values from 605 (Å)³ to 570 (Å)³ at x = 0.00 to 0.10, as detailed in Table 3.

**TABLE 3:** Different parameters of Co_0.5_Zn_0.5_La_x_ Fe_2-x_O_4_ (x = 0.00, 0.025, 0.05, 0.075, 0.10) spinel ferrites calculated from XRD data.

Concentration	0.00	0.025	0.05	0.075	0.10
Crystallite size D (nm)	17.5	22.06	24.41	25.01	26.5
Crystallite size from W-H plot	31.27	32.16	37.13	45.4	54.52
a = b = c (Å)	8.440	8.437	8.436	8.434	8.433
Unit cell Volume V (Å)^3^	601.211	600.570	600.357	599.930	599.716
X-ray density (ρ) g/cm^3^	5.38	5.42	5.55	5.68	5.75
Bulk density (ρB) g/cm^3^	3.93	4.03	4.13	4.25	4.31
Porosity (%)	26.96	25.65	25.59	25.18	25.05
Strain ε	26.60	27.87	28.14	31.28	32.67
Specific surface area S (m^2^/g)	63.72	50.18	44.29	42.24	39.38

The X-ray density (ρ_x_) and bulk density of the synthesized samples were computed using the following Equations 6, 7 (Shoba and Kaleemulla, 2017; Kalam et al., 2018):

$$\rho_{x}=\frac{nA}{VN_{A}}$$ (6)

$$\rho_{b}=\frac{m}{\pi r^{2}h}$$ (7)

where n, A, N_A_, and V represent the number of atoms in the unit cell volume, molar mass, Avogadro’s number, and unit cell volume, respectively, of the spinel lattice.

X-ray density and bulk density show a rise with La doping, attributed to the increased atomic mass of La relative to Co and Zn, influencing the overall density and consequently, structural and material properties, as illustrated in Figure 2D.

The specific values for X-ray density and bulk density for the prepared samples are indicated in Table 3. Additionally, porosity and specific surface area (S) can be calculated employing Equations 8, 9:

$$P=\left[1-\frac{\rho_{b}}{\rho_{x}}\right]\times 100$$ (8)

$$S=\frac{6000}{\rho_{x}\times D}$$ (9)

A slight reduction in porosity is noted, potentially contributing to enhanced mechanical strength and density, though careful consideration of doping levels is essential. Strain values (see Table 3) indicated the changes in lattice structure, impacting electrical and magnetic properties. Moreover, the decrease in specific surface area with increased La doping suggests reduced reactivity, a factor of significance in applications where surface properties, such as catalysis, play a crucial role. The arrangement of cations within the [A] and [B] sites significantly influenced the magnetic, structural and dielectric characteristics of ferrites (Gore et al., 2017; Islam et al., 2022). The cation distribution within ferrites can be determined using XRD data, with analysis conducted on planes such as (2 2 0), (4 0 0), (4 2 2) and (4 4 0) (Sanchez-Lievanos et al., 2021; Hasan and Azhdar, 2022). Structural factors for these (hkl) planes were computed using established equations (Gómez et al., 2018; Guo et al., 2022). Within the lattice, Zn^2+^ ions prefer occupying the (A) site, rare earth cations favour the [B] site, while both Co^2+^ and Fe^3+^ ions can occupy either [A] or [B] sites (Andhare et al., 2020; Mugutkar et al., 2020; Sanchez-Lievanos et al., 2021). Cation distribution results for Co_0.5_Zn_0.5_La_x_ Fe_2-x_O_4_ spinel ferrites are presented in Table 4.

**TABLE 4:** Cation distribution from XRD and Rietveld refinement of Co_0.5_Zn_0.5_La_x_Fe_2-x_O_4_ (x = 0.00, 0.025, 0.05, 0.075, 0.10) spinel ferrites.

Concentration	XRD	Rietveld refinement
0.00	(Zn_0.4_Fe_0.6_) [Co_0.8_Fe_1.2_]	(Zn_0.3999_Fe_0.6_) [Co_0.7999_Fe_1.2_
0.025	(Zn_0.4_Co_0.1_Fe_0.5_) [Co_0.7_Fe_1.275_La_0.025_]	(Zn_0.3998_Co_0.0999_Fe_0.4998_) [Co_0.6998_Fe_1.2749_La_0.0249_]
0.05	(Zn_0.4_Co_0.2_Fe_0.4_) [Co_0.6_Fe_1.35_La_0.05_]	(Zn_0.3998_Co_0.1997_Fe_0.3999_) [Co_0.5998_Fe_1.349_La_0.0499_]
0.075	(Zn_0.4_Co_0.3_Fe_0.3_) [Co_0.5_Fe_1.425_La_0.075_]	(Zn_0.3999_Co_0.2998_Fe_0.2999_) [Co_0.4999_Fe_1.4249_La_0.0749_]
0.10	(Zn_0.4_Co_0.4_Fe_0.2_) [Co_0.4_Fe_1.5_La_0.10_]	(Zn_0.3998_Co_0.3999_Fe_0.1999_) [Co_0.3998_Fe_1.4499_La_0.0999_]

The distribution of cations was determined through Rietveld refinement, aligning closely with the estimated values. Introducing rare earth cations at the [B] site led to a migration of Co^2+^ ions to the [A] site, accompanied by a slight shift of Fe^3+^ ions from the [A] to the [B] site. As RE (La^3+^) substitution increased in Co_0.5_Zn_0.5_La_x_ Fe_2-x_O_4_ spinel ferrites, there was a gradual rise in the proportion of Fe^3+^ ions at the [B] site, diminishing their presence at the [A] site. Utilizing equations from established sources (Gómez et al., 2018), we calculated parameters such as a_th_, r_A_, and r_B_ for [A] and [B], as detailed in Table 5. The values of a_th_ closely align with the experimentally calculated “a”, validating the predicted cation distribution.

**TABLE 5:** Theoretical parameters of Co_0.5_Zn_0.5_La_x_ Fe_2-x_O_4_ (x = 0.00, 0.025, 0.05, 0.075, 0.10).

Concentration	0.00	0.025	0.05	0.075	0.10
r_A_ (Å)	0.698	0.705	0.713	0.720	0.728
r_B_ (Å)	0.700	0.701	0.702	0.703	0.704
a_th_ (Å)	8.493	8.507	8.522	8.536	8.550
u (Å)	0.3872	0.3875	0.3877	0.3881	0.3883
δ (Å)	0.0122	0.0125	0.0127	0.0131	0.0133
R_A_ (Å)	1.8613	1.8648	1.8684	1.8723	1.8758
R_B_ (Å)	2.0242	2.0257	2.0292	2.0308	2.0360
R (Å)	6.2894	6.3037	6.3231	6.3398	6.3592
R' (Å)	5.7028	5.7103	5.7150	5.7206	5.7280
R'' (Å)	3.0076	3.0124	3.0194	3.0230	3.0313

As the RE (La^3+^) substitution increased at the [B] site, the ionic radii at that site also increased. Similarly, at the [A] site, the ionic radii rise with increasing small concentrations of Co^2+^. Consequently, both the [A] and [B] sites experience lattice expansion. An oxygen parameter, represented by “u”, helps distinguish between the expansions at [B] and [A] sites. Ideally, both sub-lattices should expand in the same ratio, with u = 3/8 = 0.375 being the expected value. In the current Co_0.5_Zn_0.5_La_x_Fe_2-x_O_4_ spinel ferrites, a value of u = 0.375 indicates significant expansion in the tetrahedral lattice, signifying the movement of oxygen ions from their ideal locations along the body diagonal of the cubic lattice (Aslam et al., 2021).

The addition of RE (La^3+^) ions into Co-Zn spinel ferrites does lead to a redistribution of cations among the 8 tetrahedral and 16 octahedral sites. The proposed cation arrangement is detailed in Table 5. In this configuration, both [A] and [B] sites are occupied by Co^2+^ and Fe^3+^ ions, while Zn^2+^ ions are specifically situated at the tetrahedral sites. Notably, due to their larger ionic radii, RE (La^3+^) ions exclusively occupy positions r_A_ and r_B_ at the [A] and [B] sites, respectively. As the RE (La^3+^) concentration increases, the value of r_B_ also increases, implying an expansion at the [B] site. The calculated a_th_ values show an increase from 8.493 to 8.550 with La^3+^ ion substitution (as seen in Table 5). This increase can be linked to the challenge of replacing smaller ferric ions with larger RE (La^3+^) ions within the lattice sites. Consequently, only a limited number of rare earth cations are retained in proximity to the grain boundaries.

This discrepancy may stem from disparities between experimental and theoretical lattice parameters. The oxygen parameter (u) was calculated using established formulas based on prior research (Gómez et al., 2018; Islam et al., 2022; Xue et al., 2022). As a result, metal ions face a more significant challenge in occupying the [A] site as opposed to the [B] site. The shifting of oxygen ions contributed to the expansion of the [A] site and the contraction of the [B] site, a phenomenon that is demonstrated by variations in the oxygen positional parameter (u). With an increase in RE (La^3+^) substitution, both the calculated u and inversion parameters experience an increase. The shifts in the calculated R_A_ and R_B_ values are contingent on the a_th_ parameter, as detailed in Table 5. Varied magnetic interactions, denoted as (A-A), (A-B) and (B-B) as explained later, result from differences in cation-cation and cation-anion bond lengths (Islam et al., 2022). The measurements of R, R′ and R″ were determined using the subsequent equation (Gore et al., 2017; Islam et al., 2022). The values of R, R′ and R″ increased by increasing the concentration of La^3+^ ion and the value of a_th_. The intensity of magnetic interactions is mostly determined by interionic distances and bond angles (Gore et al., 2017).

The calculations of the distances between cation-anion and cation-cation ions were performed using the following mathematical expressions (Gómez et al., 2018; Islam et al., 2022). When the concentration of La^3+^ ion increases the value of interionic distances also increases as given in Table 6. The bond angles θ_1_, θ_2_, θ_3_, θ_4_, and θ_5_ were determined from the equations referenced in Islam et al. (2022) and Gómez et al. (2018). A rise in the concentration of RE (La) was seen to result in decreased values for θ_1_, θ_2_, and θ_5_, indicating a reduction in the intensity of A-A and A-B interactions. Conversely, B-B interactions appeared to strengthen, as indicated by the increased values of θ_3_ and θ_4_ (refer to Table 7). The hopping lengths L_A_ and L_B_ at the A and B sites were calculated using the relationships provided in Anwar et al. (2020), and Table 7 presents the corresponding values for L_A_ and L_B_. Notably, in Co-ferrite, both L_A_ and L_B_ exhibited significant increases with the rising concentration of La^3+^.

**TABLE 6:** Cation-anion and cation-cation distances for Co_0.5_Zn_0.5_La_x_Fe_2-x_O_4_ (x = 0.00, 0.025, 0.05, 0.075, 0.10).

Concentration	0.0	0.025	0.05	0.075	0.10
p (Å)	2.0189	2.0189	2.0217	2.0257	2.0297
q (Å)	2.0175	2.0243	2.0320	2.0403	2.0480
r (Å)	3.8632	3.8763	3.8910	3.9069	3.9217
s (Å)	3.7350	3.7409	3.7517	3.7580	3.7679
b (Å)	3.0016	3.0052	3.0087	3.0158	3.0228
c (Å)	3.5197	3.5239	3.5280	3.5363	3.5446
d (Å)	3.6762	3.6806	3.6849	3.6935	3.7022
e (Å)	5.5144	5.5209	5.5274	5.5403	5.5533
f (Å)	5.1990	5.52051	5.2112	5.2235	5.2357

**TABLE 7:** Values of bond angles for Co_0.5_Zn_0.5_La_x_ Fe_2-x_O_4_ (x = 0.00, 0.025, 0.05, 0.075, 0.10).

Concentration	0.00	0.025	0.05	0.075	0.10
θ_1_	121.3657	121.2786	120.9774	120.8505	120.7437
θ_2_	136.9870	136.6271	136.0379	135.6923	135.4223
θ_3_	127.2216	127.3945	127.4305	127.4594	127.4733
θ_4_	126.3630	126.4841	126.5126	126.6481	126.6767
θ_5_	69.4209	69.1881	68.9120	68.7277	68.5801
L_A_ (Å)	3.6759	3.6794	3.6806	3.6915	3.6997
L_B_ (Å)	3.0014	3.0045	3.0118	3.0148	3.0217

### 3.2 Morphological and elemental analysis

**FIGURE 5:**
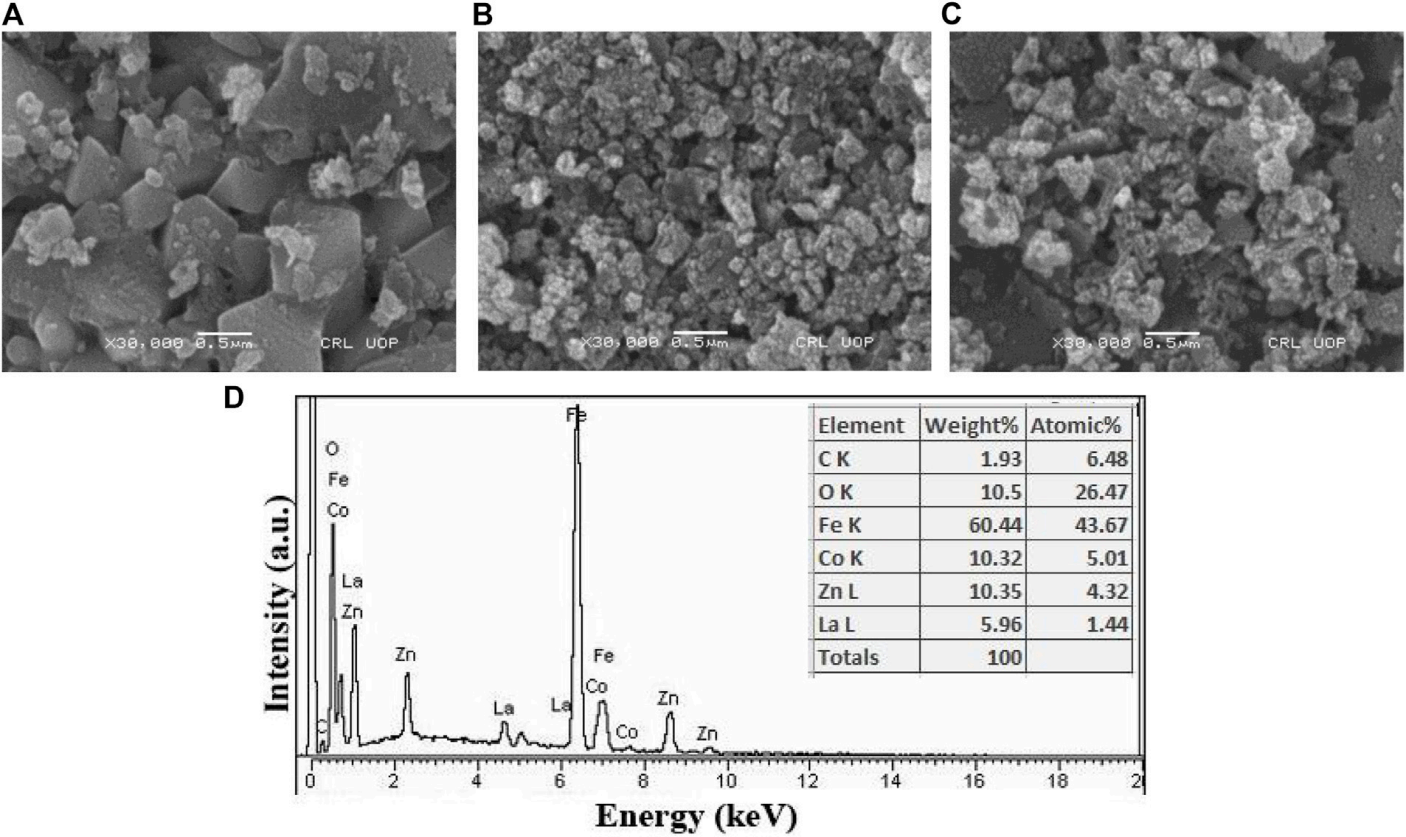
SEM images of Co_0.5_ Zn_0.5_ La_x_Fe_2-x_O_4_ at **(A)** x = 0.00 **(B)** x = 0.05 **(C)** x = 0.10 **(D)** EDX spectrum (x = 0.05).

In Figures 5A–C, the microstructures of the prepared samples with concentrations x = 0.0, 0.05 and 0.10 are presented. The micrographs revealed a uniform distribution of grains on the surface and a noticeable trend of reduction in grain size with increased lanthanum substitution. This size reduction can be attributed to the larger ionic radius of lanthanum in comparison to iron, resulting in a notable increase in particle size as the lanthanum content increased. Additionally, particle agglomeration becomes more prominent as lanthanum concentration increases (Thakur et al., 2019; Kadam et al., 2020). Energy-dispersive X-ray analysis was conducted to evaluate the elemental composition of Co_0.5_Zn_0.5_La_0.05_Fe_1.95_O_4_, as depicted in Figure 5D. The analysis confirmed the absence of contaminants in the prepared sample. Consequently, the current synthesis method has yielded mixed ferrites with a high degree of purity and excellent stoichiometry.

### 3.3 FT-IR and UV visible spectroscopy analysis

**FIGURE 6:**
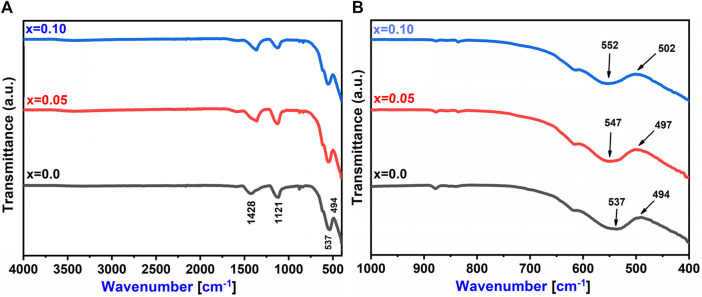
**(A)** FTIR spectra **(B)** Metal oxide bond of Co_0.5_Zn_0.5_La_x_Fe_2-x_O_4_ (x = 0.00, 0.05, 0.10).

In Figure 6A, the FTIR spectra of Co_0.5_Zn_0.5_La_x_Fe_2-x_O_4_ (x = 0.00, 0.05 and 0.10) spinel ferrites are displayed and analyzed within the range of 400–4,000 cm^−1^. Notably, two distinctive bands are observed in ferrite materials, both with widths of less than 1,000 cm^−1^, corresponding to the vibrations of the M-O bond. Upon the substitution of RE (La^3+^), the frequency bands ν_1_ and ν_2_ undergo shifts in their positions, indicating a redistribution of cations. The equation found in Mohamed and Wahba (2014), Jeevanantham et al. (2021), Ahmed et al. (2022) is utilized for the computation of the force constant (K) of ions at both tetrahedral and octahedral sites. Table 8 displays the calculated K_o_ and K_t_ values for the [B] and [A] sites, respectively. As the RE (La^3+^) substitution increased, the force constants K_t_ and K_o_ also experienced an increase, changing from 22.52 N/m to 23.26 N/m at the [A] site and from 26.62 N/m to 25.70 N/m at the [B] site (Ahmed et al., 2022).

**TABLE 8:** The values of vibrational mode frequencies (ν_1_ and ν_2_) and ’Force constants (K_t_, K_o_ and K)’ for Co_0.5_Zn_0.5_La_x_Fe_2-x_O_4_ (x = 0.00, 0.05, and 0.10) spinel ferrites.

Concentration	0.00	0.05	0.10
ν_1_ (cm^−1^)	494	497	502
ν_2_ (cm^−1^)	537	547	552
K_t_ (N/m)	22.52	22.80	23.26
K_o_ (N/m)	26.62	27.23	28.13
K	24.57	25.01	25.70

**FIGURE 7:**
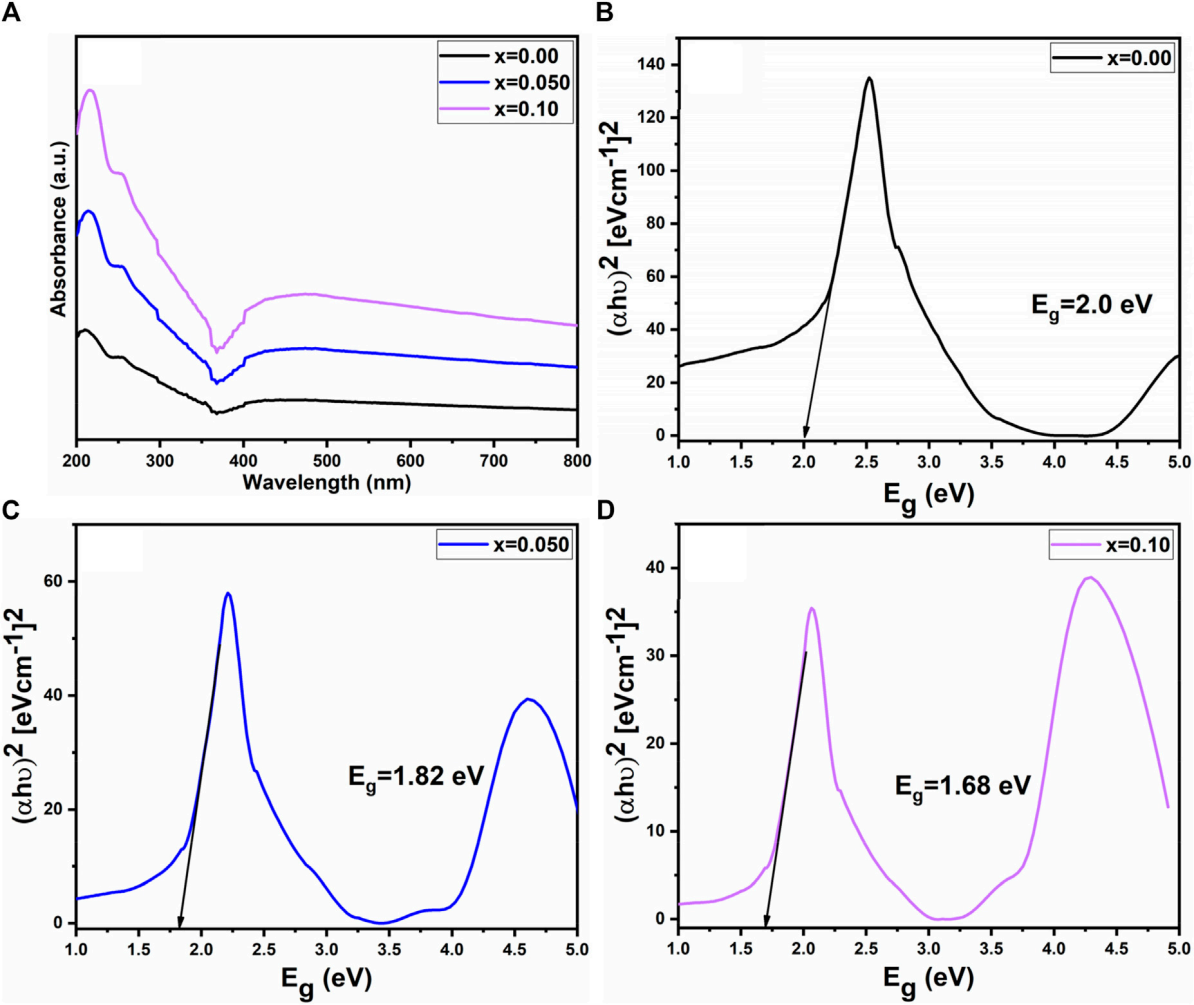
**(A)** UV visible spectra **(B–D)** Tauc plots of Co_0.5_Zn_0.5_La_x_Fe_2-x_O_4_ (x = 0.00, 0.05, 0.10).

Figure 6B further confirmed the presence of spinel ferrite structure within the synthesized samples, as the M-O bonds are detected under 1,000 cm^−1^. Notably, the absence of hydroxyl groups signifies that the prepared samples are fully dried (Hussain et al., 2022). In the ultraviolet-visible spectrum analysis of prepared samples, scanning of the ultraviolet-visible spectrum was conducted across the range of 200–800 nm illustrated in Figure 7A. It is observed that the optical energy band gap diminished as the lanthanum content in the system increased. This behavior aligns with findings from Sati et al. (2014), which indicate that the optical band gap in nano ferrites is influenced by factors such as dopant concentration, structural parameters, particle size, surface effects, lattice stresses, and contaminants. The value of the optical band gap (E_.g.,_) for the prepared samples was determined using a Tauc plot. As shown in Figures 7B–D, the energy bandgap (E_.g.,_) decreased with the rise in lanthanum substitution, ranging from 2.0 eV to 1.68 eV as x varies from 0.00 to 0.10 (Cyriac et al., 2020; Ahilandeswari et al., 2022).

The bandgap in spinel ferrites critically determines their electrical and magnetic characteristics, serving as the energy gap between the valence and conduction bands in their electronic structure. A reduced bandgap enhances electrical conductivity in these semiconducting materials, rendering them suitable for specific electronic applications. This modulation also affects the alignment of electrons, influencing magnetic properties such as magnetic moment and susceptibility, making the material more responsive to external magnetic fields. In electronic devices like magnetic sensors and microwave devices, tuning the bandgap customizes the electrical and magnetic properties of spinel ferrites to meet specific application requirements.

Furthermore, spinel ferrites with adjusted bandgaps can exhibit multifunctional behaviour, combining magnetic and semiconducting features in a single material, advantageous for advanced electronic and spintronic devices. The thermoelectric performance of spinel ferrites is impacted by bandgap modulation, suggesting potential applications in thermoelectric devices for energy conversion.

### 3.4 Dielectric analysis

**FIGURE 8:**
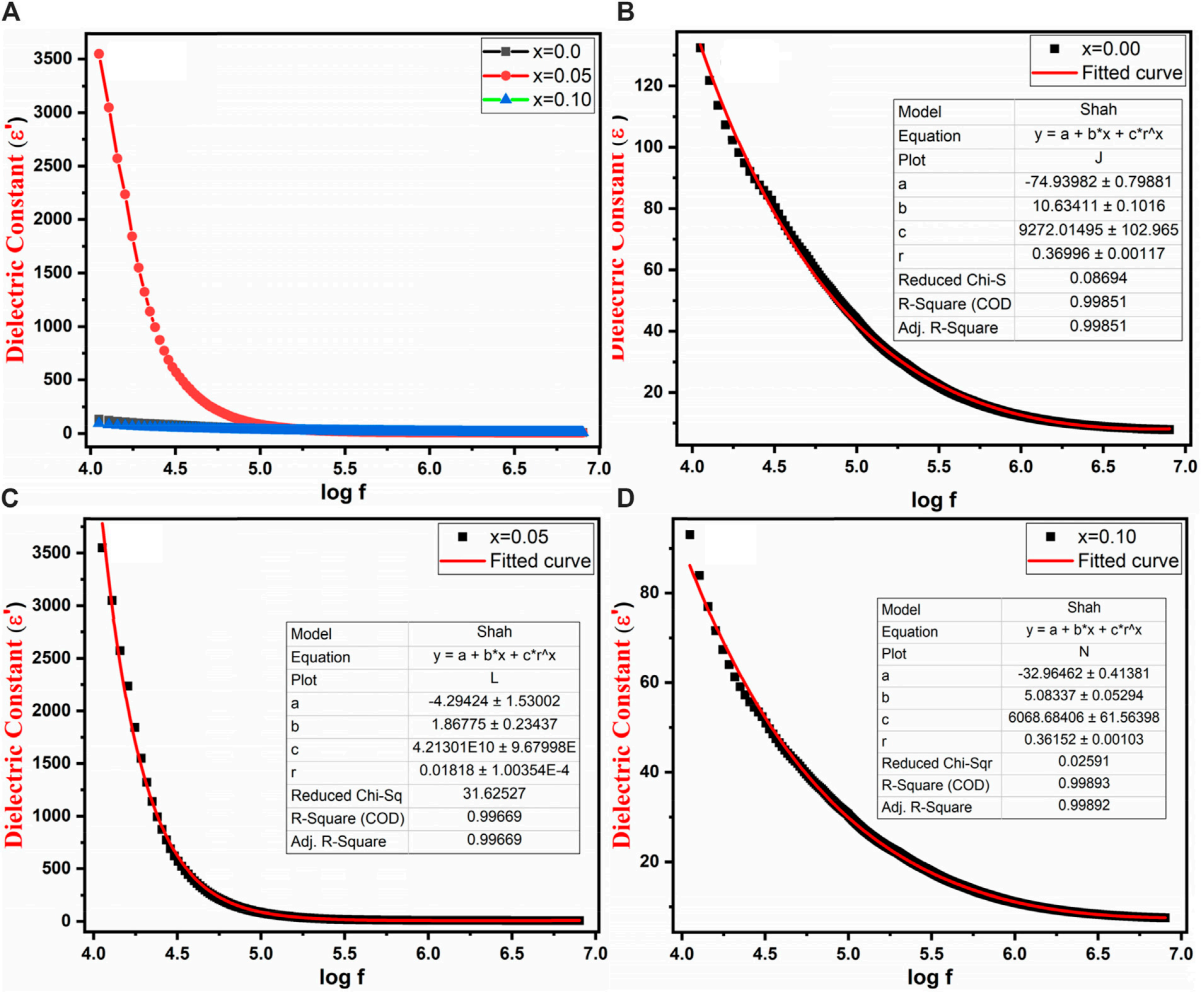
**(A)** Variation of dielectric constant with log frequency **(B–D)** Variation of dielectric constant with log frequency and Shah fitting of Co_0.5_Zn_0.5_La_x_Fe_2-x_O_4_ (x = 0.00, 0.05, 0.10).

Figure 8A illustrates the behavior of the real component of the dielectric constant (εʹ) for Co_0.5_Zn_0.5_La_x_Fe_2-x_O_4_ (x = 0.00, 0.05, 0.10) across a frequency range from 10 kHz to 8 MHz. The real component of the dielectric constant (εʹ) is indicative of the material’s ability to store electric energy. The graph reveals that at lower frequencies, the dielectric constant (εʹ) showed maximum value. Conversely, at higher frequencies, all samples exhibited a consistent behavior that remains unaffected by the applied field. This behavior is in line with the typical conduct of normal ferrites (Maksoud et al., 2020; Ansari et al., 2021; Mahmood and Maqsood, 2021). The experimental results for the dielectric constant were compared with the theoretically calculated results obtained using Shah’s function depicted in Figures 8B–D.

The relaxation phenomenon is rooted in the exchange of ions between Fe^2+^ and Fe^3+^ as well as space charge polarization. Within ferrites, the conduction mechanism of grain and grain boundaries is explained by the Maxwell-Wagner model and Koop’s hypothesis (Jnaneshwara et al., 2014; Kumari et al., 2014). Specifically, at lower frequencies, the obvious peak value of εʹ can be attributed to the impact of grain boundaries. As frequency increases, this behavior becomes independent of frequency and stabilized. The concept of space charge polarization primarily arises from the exchange of electrons between ions of the same element but with varying valence states, particularly at the B sites (Phor and Kumar, 2019). At lower frequencies, electrons within the grains may have sufficient time to move toward the grain boundaries, leading to polarization and hence higher ɛʹ at lower frequencies. However, with increasing frequency, the exchange of ions between Fe^2+^ and Fe^3+^ ions face challenges in keeping pace with the rapid oscillations of the applied field. This is due to the fact that electrons can only migrate over a fraction of a second before the field undergoes a reversal. Consequently, the window of opening for electrons to reach the grain boundary diminishes, leading to a reduced influence on polarization (Iqbal et al., 2014).

**FIGURE 9:**
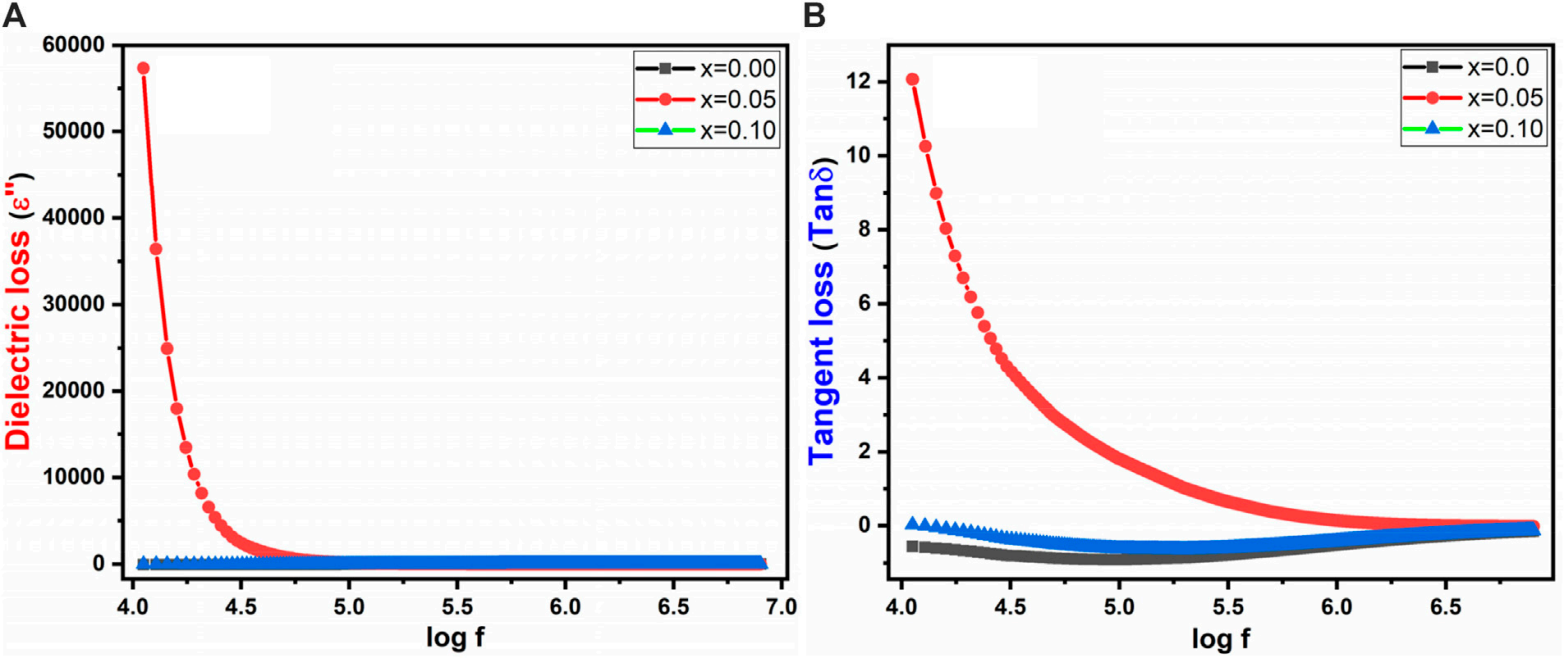
**(A)** εʹʹ **(B)** tan δ vs. log frequency of Co_0.5_Zn_0.5_La_x_Fe_2-x_O_4_ (x = 0.00, 0.05, 0.10).

Figures 9A, B depicts the dielectric loss (εʹʹ) and tangent loss of prepared spinel ferrites, respectively. The loss factor of a dielectric material is defined by Equation 10:

$$\varepsilon^{\prime \prime }=\text{Tan}\delta \varepsilon^{0}$$ (10)

Here, ε₀ represents the dielectric constant, and ε′′ denotes the dielectric loss factor. This tangent loss is contingent on the polarization arising from grain conduction, which is stimulated by the electron transfer occurring within the grain between Fe^3+^ and Fe^2+^ as well as Co^2+^ and Co^3+^.

In ferrites, the presence of electrons at grain boundaries impede inter-grain conduction. As the accumulation of electrons at boundaries intensifies, the quantity of space charge accumulates, resulting in a more pronounced potential barrier. The high surface area of nanostructured particles in cobalt zinc spinel ferrites offers a favorable environment for this phenomenon. Lower potential barriers at the grain boundaries lead to a decrease in space charge polarization when grain boundary conduction is active. Hence, inter-grain conduction prevails due to the reduced potential barrier at the grain boundary (Iqbal et al., 2014). This alteration results in a reduction in grain boundary polarizability, which is reflected as a dielectric loss. It is worth noting that tanδ demonstrates a higher value at lower frequencies and a lower value at higher frequencies, primarily due to the increased responsiveness of space charge polarization at lower frequencies (as shown in Figure 9B). Spinel ferrites are a distinct class of magnetic materials renowned for their interesting magnetic and dielectric behavior. This comprehension is important for varied applications, particularly within electronics and telecommunications. The tangent loss, also known as the dissipation factor or loss tangent, measures the amount of energy converted into heat during each cycle of an electric field within a dielectric material. Its expression as the ratio of the imaginary to the real part of complex permittivity underscores its sensitivity to distinct material phenomena or transitions, thereby contributing to a better understanding of spinel ferrites’ behavior.

**FIGURE 10:**
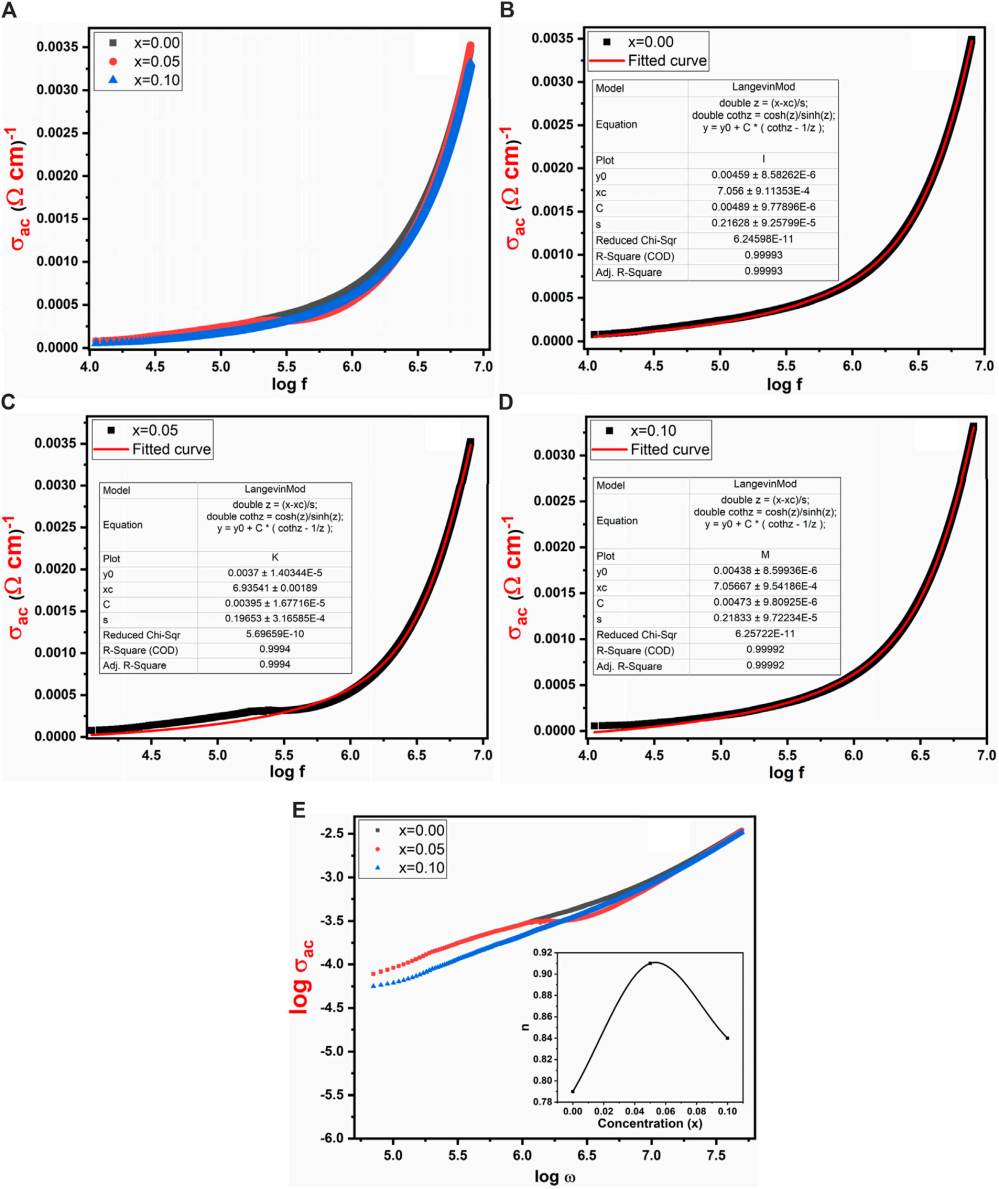
Frequency verses **(A)** σ_ac_ **(B–D)** AC conductivity with curve fitting by using Langevin Mod fitting **(E)** log σ_ac_ vs. log ω of Co_0.5_Zn_0.5_La_x_Fe_2-x_O_4_ (x = 0.00, 0.05, 0.10), inset: frequency exponent “n” vs. concentration.

Figure 10A investigated the influence of frequency range from 10 kHz to 8 MHz on conductivity (σ_ac_) for Co_0.5_ Zn_0.5_ La_x_Fe_2-x_O_4_ (x = 0.00, 0.05, 0.10). The power of conduction within the material can be assessed through its conductivity (σ_ac_). Figure 10A shows that AC conductivity exhibits a low value at low frequencies but rises rapidly at higher frequencies. This behavior aligns with the Double-Layer Maxwell and Wagner model, which suggests that at low frequencies, grain boundaries mainly influence conductivity, whereas at higher frequencies, grain activity becomes more distinct, facilitating electron hopping processes. In the present study, at higher frequencies, conductivity significantly increases due to the involvement of bounded charge carriers in the conductivity mechanism (Iqbal et al., 2014). Additionally, Figure 10A reveals two distinct frequency regimes: a plateau region corresponding to low frequencies, where conductivity remains independent of frequency, and a dispersion region corresponding to high frequencies, where conductivity escalates with increasing frequency (Chahar et al., 2022). The experimental findings regarding the conductivity (σ_ac_) were contrasted with the theoretically computed outcomes derived from Langevin Mod’s function, as illustrated in Figures 10B–D. The exponent “n” can be computed using the given relation (Equation 11) by plotting graphs between log σ_ac_ and log ω shown in Figure 10E:

$$\sigma \left(\omega \right)=B\times \omega^{n}$$ (11)

The relationship between log σ_ac_ and log ω shows a consistent pattern for x = 0.0, where the slope corresponds to the exponent “n” and the intercept aligns with log B at log ω = 0. Importantly, it is notable that “n” falls within the range of 0–1. When “n” equals 0, electrical conduction remains unaffected by frequency, whereas “n” values less than or equal to 1 signify frequency-dependent electrical conduction. In this study, “n” ranges from 0.79 to 0.84, indicating that the samples exhibit frequency-dependent behavior.

**FIGURE 11:**
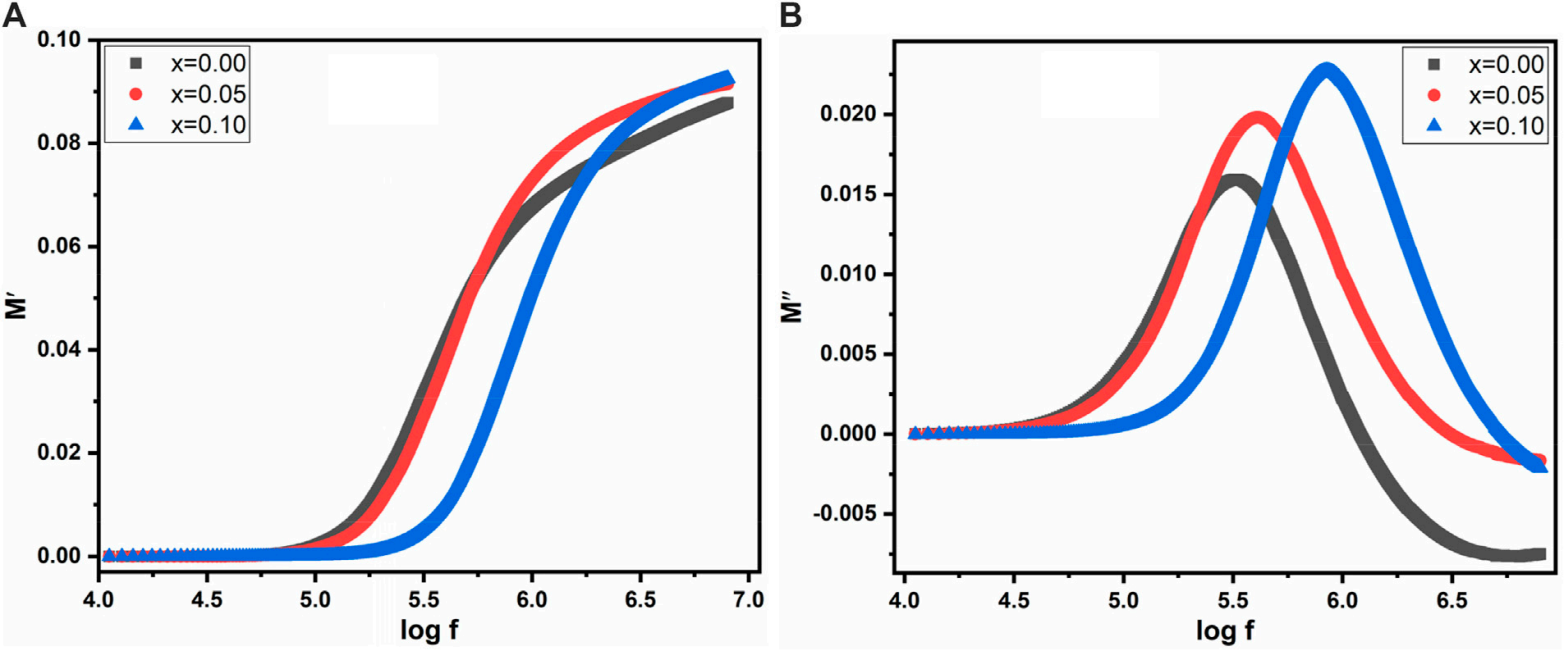
Frequency verses **(A)** M′ **(B)** M″ for Co_0.5_Zn_0.5_La_x_Fe_2-x_O_4_ (x = 0.00, 0.05, 0.10).

The graph in Figure 11A illustrates the variation of electric modulus (M′) with the log of frequency across all samples. The total electrical modulus “M” comprises contributions from both the real and complex moduli, represented by the following Equation 12:

$$M_{\text{total}}=M^{\prime }+{\text{jM}}^{\prime \prime }$$ (12)

In the above Equation 12, the first factor, M′, can be determined using the following expression (Equation 13):

$$M^{\prime }=\frac{\varepsilon^{\prime }}{{\varepsilon^{\prime }}^{2}+{\varepsilon^{\prime \prime }}^{2}}$$ (13)

Similarly, the second factor in Equation 12, M″, can be calculated using Equation 14:

$$M^{\prime \prime }=\frac{\varepsilon^{\prime \prime }}{{\varepsilon^{\prime }}^{2}+{\varepsilon^{\prime \prime }}^{2}}$$ (14)

The electrical modulus exhibits exciting behavior with changing frequency. Figure 11A reveals that at lower frequencies, the electric modulus maintains a constant value, attributed to electrode effects or ionic polarization. However, at higher frequencies, all samples (with x = 0.00, 0.05 and 0.10) demonstrated significant asymmetric behavior, indicative of space charge polarization in the high-frequency region.

Figure 11B displays the behavior of the complex modulus, M″, against the log of frequency. At high frequencies, a distinct sharp peak is observed across all samples, reflecting maximum asymmetric tendencies.

This peak signifies relaxation behavior and is associated with the single polarization phenomenon of grain boundaries. Moreover, with the substitution of La^3+^, the peak intensity increases, and there is a slight shift towards higher frequencies, indicating the presence of dielectric relaxation within the material. Furthermore, the trend observed in the M″ graph at low frequencies suggests that charge carriers undergo significant displacement over larger distances, whereas at high frequencies, their mobility is constrained to shorter distances. This alteration in charge carrier behavior with frequency variations across all samples suggests a shift from small to longer-range mobility, revealing insights into the material’s conductivity properties (Mustafa et al., 2022).

#### 3.4.1 Cole-Cole

**FIGURE 12:**
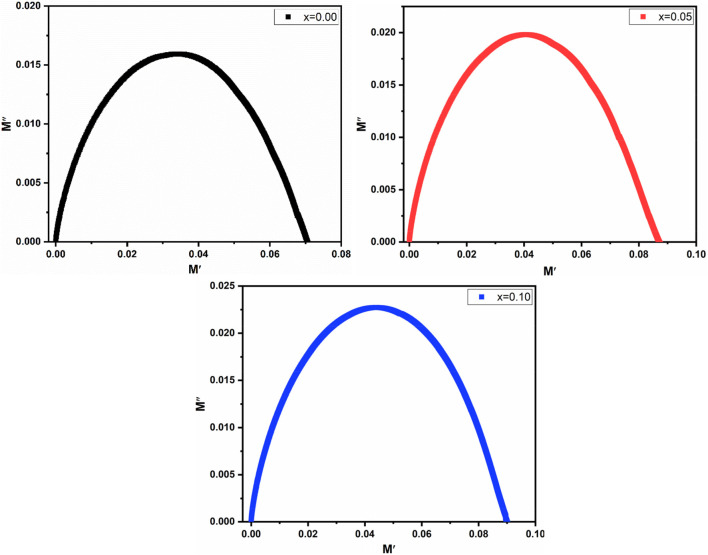
Cole-Cole plot of Co_0.5_ Zn_0.5_ La_x_Fe_2-x_O_4_ (x = 0.00, 0.05, 0.10).

Figure 12 depicts the Cole-Cole plot representing the complex modulus plane (M′ vs. M″). The Cole-Cole plot serves as a valuable tool for investigating the conduction mechanisms within both grains and grain boundaries.

M′ and M″ were chosen for the Cole-Cole plot as alternative methods did not yield suitable results (Dhabekar and Kant, 2021). The semicircles evident in the Cole-Cole plots provide valuable insights into the material’s conducting capacity. Figure 12 demonstrates that only one semicircle is consistently observed in all samples within the Cole-Cole plot. This observation elucidates the reason for the low grain boundary resistance in the high-frequency region (Pandit et al., 2014). The La^3+^ substitution has different effects on grain and grain boundary resistance, as seen by the shift in the radius of the semicircle. According to Dewi et al. (2023), the change in radius might be caused by the distortion of the lattice that occurs because of increasing La^3+^ substitution and attributed to the fluctuation in the relaxation frequency. It can be seen that at x = 0.05 to x = 0.10, the hight of the semicircle changes due to polarization relaxation processes that are caused by the change of field in the ferrites (Junaid et al., 2016).

### 3.5 Magnetic properties

**FIGURE 13:**
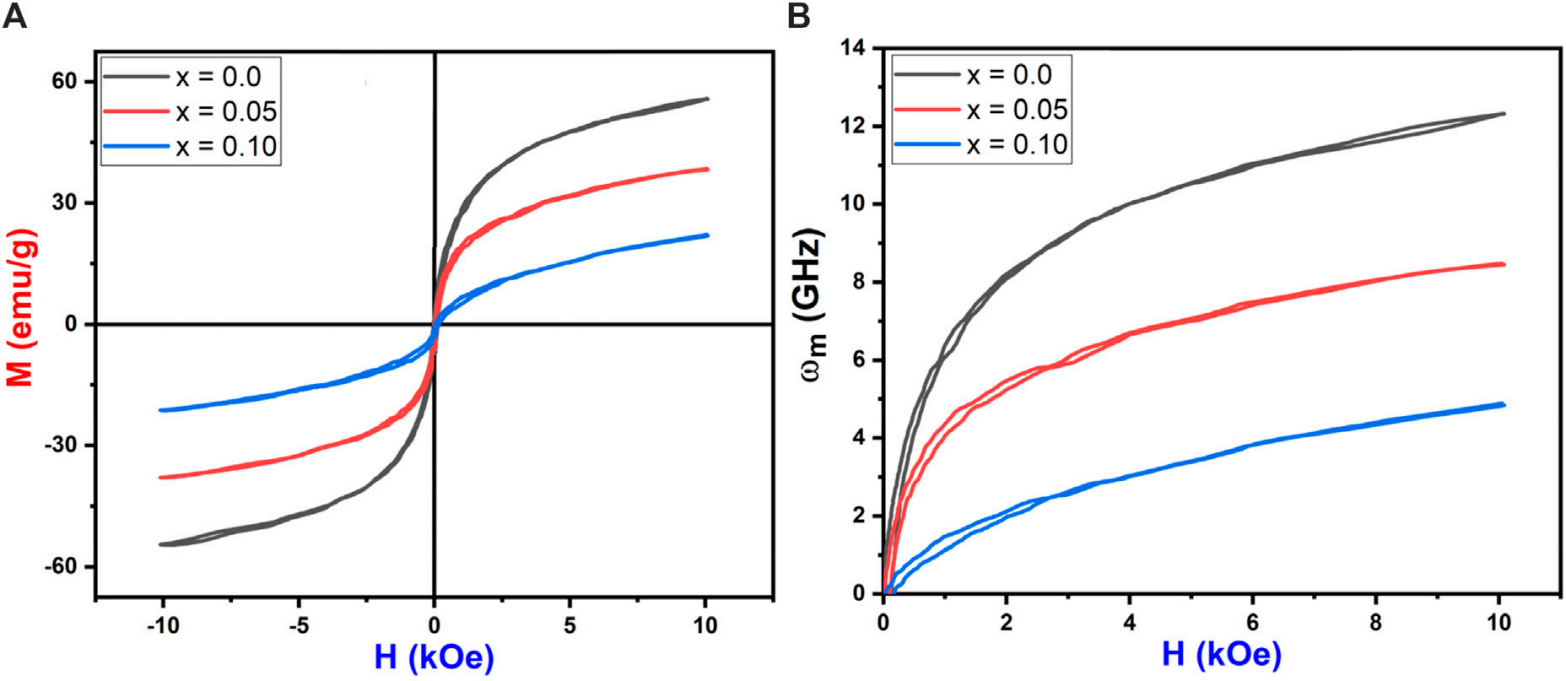
**(A)** M-H loops **(B)** Applied field vs. microwave frequency of Co_0.5_Zn_0.5_La_x_Fe_2-x_O_4_ (x = 0.00, 0.05, 0.10).

The M-H loops of Co_0.5_Zn_0.5_La_x_Fe_2-x_O_4_ (x = 0.0, 0.050, 0.10) ferrite powders at room temperature were recorded using a VSM (see Figure 13A). The loops in Figure 13A illustrate that the as-synthesized samples exhibit small areas (narrow loops), indicating soft magnetic behavior (Datta et al., 2023). The saturation magnetization (Ms) values for the Co_0.5_Zn_0.5_La_x_Fe_2-x_O_4_ ferrite samples were observed to decrease from 55.84 emu/g to 22.08 emu/g as the concentration of La_3+_ ion increased. From Table 9, it is evident that the saturation magnetization (M_S_) decreases with the doping of La^3+^ rare earth ions. Typically, when Fe^3+^ ions in ferrites are replaced by RE^3+^ ions with smaller magnetic moments, the magnetization of RE^3+^-doped ferrites decreases. Conversely, if the magnetic moment of the RE^3+^ ion exceeds that of Fe^3+^, the magnetization increases. However, this behavior is not consistent across all RE-doped ferrites. It is reported that rare earth ions, when doped in ferrites, prefer to occupy the octahedral B site (Nikumbh et al., 2014; Ghosh et al., 2020; Ahmed et al., 2022). The Bohr magnetron and magnetic moment of prepared NPs were calculated from the following Equations 15, 16:

$$\mu_{B}=\frac{M_{s}\times M_{w}}{5585\times dx}$$ (15)

$$n_{B}=\frac{M_{s}\times M_{w}}{5585}$$ (16)

where (Ms) is magnetization saturation, (d_x_) is X-ray density and (M_w_) is molecular mass. It is observed that the magneton number value decreased with increasing concentration (x) presented in Table 9.

**TABLE 9:** Magnetic parameters and cation distribution of Co_0.5_Zn_0.5_La_x_Fe_2-x_O_4_ (x = 0.00, 0.05, 0.10).

x	0.00	0.05	0.10
Ms (emu/g)	55.84	38.31	22.08
Mr (emu/g)	2.08	0.75	0.795
Hc (Oe)	25.63	13.33	33.88
SQ (Mr/Ms)	0.037	0.019	0.036
ω_m_ (GHz)	12.3	8.5	4.9
K (erg/cm^3^)	1,490	531	799
n_B_	0.442	0.429	0.414
μ_i_	36.6	67.3	16.6
μ_B_	2.3779	2.3809	2.3805
Cation distribution	(Zn_0.3_Fe_0.7_) [Co_0.5_Fe_1.5_]	(Zn_0.3_Co_0.2_Fe_0.5_) [Co_0.3_Fe_1.65_La_0.05_]	(Zn_0.3_Co_0.4_Fe_0.3_) [Co_0.1_Fe_1.8_La_0.10_]

The decrease in magnetic moment results from the reduction in Ms. The incorporation of non-magnetic La^3+^ ions into the ferrite lattice reduces the number of magnetic ions, leading to a smaller magnetic moment. The A-B superexchange interaction, which is much stronger than the A-A and B-B interactions, exists among these interstitial sites (Dipesh et al., 2016). The remanence magnetization (M_r_) and coercivity field (H_c_) were measured from each sample’s M-H loop, as shown in Table 9. The irregular trends in M_r_ and H_c_ are due to the complex magnetic interactions in the ferrite structure influenced by the addition of La^3+^ ions. The competition between exchange interactions and anisotropy effects leads to non-linear behavior. This irregularity in coercivity and remanence magnetization indicates the material’s potential for use in magnetic recording media, where precise control of coercivity is crucial for data storage (Alzoubi, 2022).

Further more, the substitution of paramagnetic rare earth cations lead to the formation of a non-magnetic spinel structure. The magnetic moments for Zn^2+^ (0 μB), Co^2+^ (3 μB), Fe^3+^ (5 μB) (Aguilera-Del-Toro et al., 2021) and La^3+^ (0 μB) (Zhang et al., 2019) were considered in this analysis. In the case of Zn–Co ferrites doped with RE (La^3+^), La^3+^ (0 μB) replaces Fe^3+^ (5 μB) cations at the B sublattice, reducing the octahedral sublattice magnetization (M_B_). However, the tetrahedral sublattice magnetization (M_A_) remains unchanged due to the consistent cation distribution at the A-site, resulting in a decrease in the net magnetic moment (n_B_). The crystallite size (D) increases with decreasing magnetization saturation (Ms), indicating a superparamagnetic behavior. Notably, Co_0.5_Zn_0.5_La_0.1_Fe_1.9_O_4_ contains impurities such as LaFeO_3_ at grain boundaries, causing internal stress that affects the magnitude of saturation magnetization (Bharti et al., 2021).

The squareness ratio (R_sq_) was evaluated by the formula (Equation 17):

$$R_{\text{sq}}=\frac{M_{r}}{M_{s}}$$ (17)

All prepared samples exhibited R_sq_ values below 1, indicating super-paramagnetic behavior. This suggests that the magnetic moments within the samples are unaligned, switching rapidly and randomly under an external magnetic field. Such behavior is typical in nanoscale materials, likely due to the small particle size and compositional variations in the ferrite samples (Suo et al., 2020). This super-paramagnetism makes these materials suitable for targeted drug delivery using magnetic nanoparticles, as the rapid switching allows the controlled release of therapeutic agents. Additionally, spinel ferrites can be used in magnetic sensors for detecting weak magnetic fields, valuable for geophysical exploration and non-destructive testing. The anisotropy constant (K) was calculated using the relation (Equation 18):

$$K=\frac{H_{c}\times M_{s}}{0.96}$$ (18)

The smaller anisotropy constant was 531 for x = 0.05. The anisotropy constant (K) values for varying La^3+^ ion concentrations in ferrite samples were found to be 1,490, 531, and 799 erg/cm^3^ for x = 0.0, 0.05, and 0.10, respectively. This irregular trend in K is due to changes in structural and compositional features as La^3+^ is incorporated. Anisotropy in magnetic materials depends on factors such as crystallographic orientation and domain structure, which are affected by La^3+^ concentration. These variations in K can influence the materials’ effectiveness as microwave absorbers for electromagnetic interference (EMI) shielding, making them suitable for use in electronics and telecommunications. The tunable magnetic properties of these spinel ferrites provide versatility for various technological applications (Algarou et al., 2020; Bilal et al., 2024). The initial permeability (µ_i_) was estimated using Equation 19 and is reported in Table 9.

$${µ}_{i}=\frac{M_{s}^{2}\times D}{K}$$ (19)

The initial permeability (µ_i_) exhibited an irregular trend with increasing La^3+^ ion concentration, showing values of 36.6, 67.3 and 16.6 for La^3+^ concentrations of x = 0.0, 0.05 and 0.1, respectively.

**FIGURE 14:**
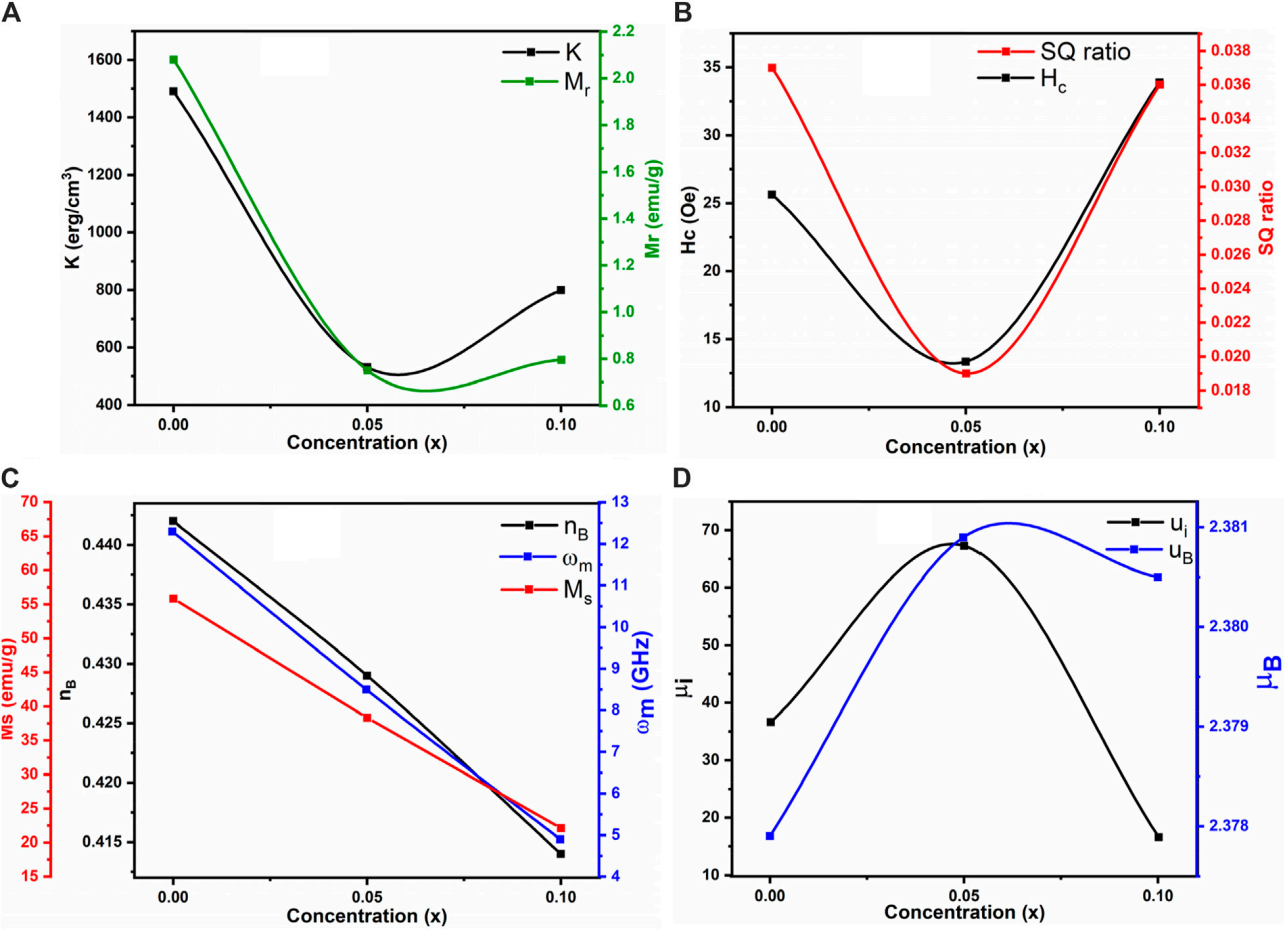
Concentration (x) vs. **(A)** Anisotropy constant and retentivity **(B)** Coercivity and remanent ratio **(C)** Saturation magnetization, Bohr magnetron and microwave frequency **(D)** Initial permeability and magnetic moment of all the samples.

This non-linear behavior can be attributed to the complex interplay of magnetic interactions within the ferrite structure, influenced by the addition of La^3+^ ions. The competition between exchange interactions and anisotropy effects contributes to this trend. The microwave frequency (ω_m_) is determined using the formula ω_m_ = 8π2M_s_γ, where γ is the gyromagnetic ratio (2.8 MHz/Oe). This indicates that higher saturation magnetization (M_s_) results in a higher microwave frequency. Figure 13B shows the relationship between the applied magnetic field and microwave frequency. High resistivity and low-loss ferrites are suitable for microwave devices (Ansari et al., 2020). The calculated operating microwave frequencies (ω_m_) for samples with x = 0.00, 0.05 and 0.1 falls within the 12–4 GHz range, making them ideal for X-band microwave applications, as detailed in Table 9. The graphical representation of La^3+^ concentration (x) versus magnetic parameters is depicted in Figures 14A–D.

## 4 Conclusion

Rare earth (La) substituted Co-Zn spinel ferrites were successfully prepared by the sol-gel auto-combustion method. The X-ray diffraction (XRD) pattern demonstrated that the synthesized spinel ferrites possess a single-phase cubic structure which was also confirmed by Rietveld refinement having crystallite size ranging from 17.5 to 26.5 nm. The substitution of lanthanum indicated a decreased behavior in lattice constant from 8.440 (Å) to 8.433 (Å), decreased in volume from 601.2 (Å)^3^ to 599.7 (Å)^3^ and increased in X-ray density from 5.38 to 5.75 g/cm^3^. Two vibrational bands, a low band (490 cm^−1^) and a high band (540 cm^−1^) confirmed by FTIR spectra. UV–vis revealed that the minimum value of the optical band gap observed was 1.68 eV for composition x = 0.10. Dielectric constant and Dielectric loss showed the same behaviour for all samples and raised with doping. AC conductivity’s value increased with doping. The dielectric tangent loss showed constant behaviour at higher frequencies for all prepared samples and had a maximum value of 12 at lower frequencies. The VSM results showed that magnetization saturation decreased from 55.84 emu/g to 22.08 emu/g and the value of coercivity increased as rare earth (La) doping increased. The microwave frequency was 4.9 GHz for x = 0.10. Therefore, such materials can be suitable for use in microwave and energy storage devices.

## Data Availability

The raw data supporting the conclusions of this article will be made available by the authors, without undue reservation.

## References

[B1] Abbas B. Ahmad A. u. Shabbir S. Shahid M. Ahmad T. Braga M. H. (2023). Enhancing photocatalytic and antibacterial performance through compositional optimization of NiO-CdO heterogeneous nanocomposites. Ceram. Int. 49, 33525–33536. 10.1016/j.ceramint.2023.07.250

[B2] Abdellatif M. H. El-Komy G. M. Azab A. A. Salerno M. (2018). Crystal field distortion of La^3+^ ion-doped Mn-Cr ferrite. J. Magn. Magn. Mat. 447, 15–20. 10.1016/j.jmmm.2017.09.040

[B3] Aguilera-Del-Toro R. H. Aguilera-Granja F. Torres M. B. Vega A. (2021). Relation between structural patterns and magnetism in small iron oxide clusters: reentrance of the magnetic moment at high oxidation ratios. Phys. Chem. Chem. Phys. 23, 246–272. 10.1039/d0cp03795h 33325468

[B4] Ahilandeswari E. Sakthipandi K. Kanna R. R. Hubálovská M. Vigneswaran D. (2022). Lanthanum substitution effect on the structural, optical, and dielectrical properties of nanocrystalline BaFe_2_O_4_ ferrites. Phys. B Condens. Matter. 635, 413849. 10.1016/j.physb.2022.413849

[B5] Ahmed I. Mustafa G. Umair Subhani M. Hussain G. Ismail A. G. Anwar H. (2022). A detailed investigation of lanthanum substituted bismuth ferrite for enhanced structural, optical, dielectric, magnetic and ferroelectric properties. Results Phys. 38, 105584. 10.1016/j.rinp.2022.105584

[B6] Ahmed I. Naz I. Morley N. Shabbir S. Maraj M. Ismail A. G. (2023). Experimental and DFT investigation of structural and optical properties of lanthanum substituted bismuth ferrites. Phys. B Condens. Matter. 661, 414927. 10.1016/j.physb.2023.414927

[B7] Algarou N. A. Slimani Y. Almessiere M. Alahmari F. Vakhitov M. Klygach D. (2020). Magnetic and microwave properties of SrFe_12_O_19_/MCe_0.04_Fe_1.96_O_4_ (M = Cu, Ni, Mn, Co and Zn) hard/soft nanocomposites. J. Mat. Res. Technol. 9, 5858–5870. 10.1016/j.jmrt.2020.03.113

[B8] Altarawneh A. M. Arrasheed E. A. Ajlouni A. W. Ghazy R. Hemeda O. Henaish A. (2023). Correlation between structural, cation distribution with dielectric spectra and magnetic properties for Co–Zn ferrite doped with La^3+^ ions. Ceram. Int. 49, 14215–14224. 10.1016/j.ceramint.2023.01.008

[B9] Alzoubi G. M. (2022). The effect of Co-doping on the structural and magnetic properties of single-domain crystalline copper ferrite nanoparticles. Magnetochemistry 8, 164–169. 10.3390/magnetochemistry8120164

[B10] Andhare D. D. Patade S. R. Kounsalye J. S. Jadhav K. M. (2020). Effect of Zn doping on structural, magnetic and optical properties of cobalt ferrite nanoparticles synthesized via. Co-precipitation method. Phys. B Condens. Matter. 583, 412051. 10.1016/j.physb.2020.412051

[B11] Ansari A. A. Abushad M. Arshad M. Naseem S. Ahmed H. Husain S. (2021). Microstructure, optical and dielectric properties of cobalt-doped zinc ferrite nanostructures. J. Mat. Sci. Mat. Electron. 32, 21988–22002. 10.1007/s10854-021-06647-2

[B12] Ansari M. M. N. Khan S. Ahmad N. (2020). Structural, electrical transport and magnetic properties of Nd^3+^ substituted Mn–Cu nanoferrites. J. Alloys Compd. 831, 154778. 10.1016/j.jallcom.2020.154778

[B13] Anwar A. Zulfiqar S. Yousuf M. A. Ragab S. A. Khan M. A. Shakir I. (2020). Impact of rare earth Dy^3+^ cations on the various parameters of nanocrystalline nickel spinel ferrite. J. Mat. Res. Technol. 9, 5313–5325. 10.1016/j.jmrt.2020.03.057

[B14] Asghar Yousaf M. I. Shad N. A. Sajid M. M. Afzal A. M. Javed Y. (2022). Enhanced electrochemical performance of hydrothermally synthesized NiS/ZnS composites as an electrode for super-capacitors. J. Clust. Sci. 33, 2325–2335. 10.1007/s10876-021-02157-7

[B15] Aslam A. Rehman A. U. Amin N. Ajaz un Nabi M. Abdullah Q. u. a. Morley N. (2021). Lanthanum doped Zn_0.5_Co_0.5_La_x_Fe_2−x_O_4_ spinel ferrites synthesized via co-precipitation route to evaluate structural, vibrational, electrical, optical, dielectric and thermoelectric properties. J. Phys. Chem. Solids. 154, 110080. 10.1016/j.jpcs.2021.110080

[B16] Bharti M. K. Chalia S. Thakur P. Thakur A. (2021). Effect of lanthanum doping on microstructural, dielectric and magnetic properties of Mn_0.4_Zn_0.6_Cd_0.2_La_x_Fe_1.8-x_O_4_ (0.0 ≤ x ≤ 0.4). J. Supercond. Nov. Magn. 34, 2591–2600. 10.1007/s10948-021-05908-9

[B17] Bilal M. Ahmed I. Shabbir S. Subhani M. U. Maraj M. Anwar H. (2024). Structural, optical, dielectric and magnetic properties of Cd substituted copper strontium W-type hexaferrite. Ceram. Int. 50, 17228–17241. 10.1016/j.ceramint.2024.02.198

[B18] Chahar D. Thakur P. Kumar R. Thakur A. (2022). Influence of Mg doping on the structural, electrical and dielectric properties of Co-Zn nanoferrites. J. Magn. Magn. Mat. 544, 168726. 10.1016/j.jmmm.2021.168726

[B19] Cyriac J. Augustine S. Kalarikkal N. Mukherjee S. Ahmed M. Nambissan P. M. G. (2020). Dysprosium-substitution-induced structural changes of multiferroic nanocrystalline bismuth ferrite and the investigation through positron annihilation and other studies. Phys. B Condens. Matter. 599, 412431. 10.1016/j.physb.2020.412431

[B20] Datta S. Manglam M. K. Panda S. K. Shukla A. Kar M. (2023). Investigation of crystal structure and magnetic properties in magnetic composite of soft magnetic alloy and hard magnetic ferrite. Phys. B Condens. Matter. 653, 414675. 10.1016/j.physb.2023.414675

[B21] Dewi S. H. Mulyawan A. Sarwanto Y. Winatapura D. S. Adi W. A. (2023). Effect of La^3+^ substitution on structural, microstructure, magnetic properties, and microwave absorbing ability of yttrium iron garnet. J. Rare Earth. 41, 578–587. 10.1016/j.jre.2022.03.003

[B22] Dhabekar K. Kant K. M. (2021). Structural and dielectric properties of cobalt ferrite based nanocomposites. Phys. B Condens. Matter. 603, 412752. 10.1016/j.physb.2020.412752

[B23] Dipesh D. N. Wang L. Adhikari H. Alam J. Mishra S. R. (2016). Influence of Al^3+^ doping on structural and magnetic properties of CoFe_2-x_Al_x_O_4_ Ferrite nanoparticles. J. Alloys Compd. 688, 413–421. 10.1016/j.jallcom.2016.07.030

[B24] Dippong T. Mereu R. A. (2024). Effect of La^3+^ on thermal, structural and morphological properties of Zn–Co ferrite spinel-based pigments. Ceram. Int. 50, 10314–10324. 10.1016/j.ceramint.2023.12.343

[B25] Gaba S. Kumar A. Rana P. S. Arora M. (2018). Influence of La^3+^ ion doping on physical properties of magnesium nanoferrites for microwave absorption application. J. Magn. Magn. Mat. 460, 69–77. 10.1016/j.jmmm.2018.03.035

[B26] Ghosh M. P. Sharma S. Satyapal H. K. Tanbir K. Singh R. K. Mukherjee S. (2020). Tuning the microstructural, optical and superexchange interactions with rare earth Eu doping in nickel ferrite nanoparticles. Mat. Chem. Phys. 241, 122383. 10.1016/j.matchemphys.2019.122383

[B27] Gilani Z. A. Farooq A. Asghar N. H. K. Khalid M. (2020). Synthesis and characterization of lanthanum doped Co-Zn spinel ferrites nanoparticles by sol-gel auto combustion method. J. Mat. Phys. Sci. 1, 1–11. 10.52131/jmps.2020.0101.0001

[B28] Gómez C. A. P. Meneses C. A. B. Matute A. (2018). Structural parameters and cation distributions in solid state synthesized Ni-Zn ferrites. Mat. Sci. Eng. B Solid-State Mat. Adv. Technol. 236-237, 48–55. 10.1016/j.mseb.2018.12.003

[B29] Gore S. K. Jadhav S. S. Tumberphale U. B. Shaikh S. M. Naushad M. Mane R. S. (2017). Cation distribution, magnetic properties and cubic-perovskite phase transition in bismuth-doped nickel ferrite. Solid State Sci. 74, 88–94. 10.1016/j.solidstatesciences.2017.10.009

[B30] Guo H. S. Zhang L. Yan Y. L. Zhang J. Wang J. Wang S. Y. (2022). Effect of lanthanum substitution on structural, magnetic, and electric properties of Ni–Zn–Co ferrites for radio frequency and microwave devices. Ceram. Int. 48, 22516–22522. 10.1016/j.ceramint.2022.04.275

[B31] Haque S. U. Saikia K. K. Murugesan G. Kalainathan S. (2017). A study on dielectric and magnetic properties of lanthanum substituted cobalt ferrite. J. Alloys Compd. 701, 612–618. 10.1016/j.jallcom.2016.11.309

[B32] Hasan S. Azhdar B. (2022). Synthesis of nickel-zinc ferrite nanoparticles by the sol-gel auto-combustion method: study of crystal structural, cation distribution, and magnetic properties. Adv. Condens. Matter Phys. 1, 1–14. 10.1155/2022/4603855

[B33] Hussain G. Ahmed I. Rehman A. U. Subhani M. U. Morley N. Akhtar M. (2022). Study of the role of dysprosium substitution in tuning structural, optical, electrical, dielectric, ferroelectric, and magnetic properties of bismuth ferrite multiferroic. J. Alloys Compd. 919, 165743. 10.1016/j.jallcom.2022.165743

[B34] Iqbal M. A. Islam M. U. Ali I. Khan M. A. Sadiq I. Ali I. (2014). High frequency dielectric properties of Eu^3+^-substituted Li-Mg ferrites synthesized by sol-gel auto-combustion method. J. Alloys Compd. 586, 404–410. 10.1016/j.jallcom.2013.10.066

[B35] Islam M. A. Hossain A. K. M. A. Ahsan M. Z. Bally M. A. A. Ullah M. S. Hoque S. M. (2022). Structural characteristics, cation distribution, and elastic properties of Cr^3+^ substituted stoichiometric and non-stoichiometric cobalt ferrites. RSC Adv. 12, 8502–8519. 10.1039/d1ra09090a 35424790 PMC8985153

[B36] Jeevanantham B. Song Y. Choe H. Shobana M. K. (2021). Structural and optical characteristics of cobalt ferrite nanoparticles. Mat. Lett. x. 12, 100105. 10.1016/j.mlblux.2021.100105

[B37] Jnaneshwara D. M. Avadhani D. Daruka Prasad B. Nagabhushana B. Nagabhushana H. Sharma S. (2014). Effect of zinc substitution on the nanocobalt ferrite powders for nanoelectronic devices. J. Alloys Compd. 587, 50–58. 10.1016/j.jallcom.2013.10.146

[B38] Junaid M. Khan M. A. Iqbal F. Murtaza G. Akhtar M. N. Ahmad M. (2016). Structural, spectral, dielectric and magnetic properties of Tb–Dy doped Li-Ni nano-ferrites synthesized via micro-emulsion route. J. Magn. Magn. Mat. 419, 338–344. 10.1016/j.jmmm.2016.06.043

[B39] Kadam A. B. Mande V. K. Kadam S. B. Kadam R. H. Shirsath S. E. Borade R. B. (2020). Influence of gadolinium (Gd^3+^) ion substitution on structural, magnetic and electrical properties of cobalt ferrites. J. Alloys Compd. 840, 155669. 10.1016/j.jallcom.2020.155669

[B40] Kalam A. Al-Sehemi A. G. Assiri M. Du G. Ahmad T. Ahmad I. (2018). Modified solvothermal synthesis of cobalt ferrite (CoFe_2_O_4_) magnetic nanoparticles photocatalysts for degradation of methylene blue with H_2_O_2_/visible light. Results Phys. 8, 1046–1053. 10.1016/j.rinp.2018.01.045

[B41] Kiani M. N. Butt M. S. Gul I. H. Saleem M. Irfan M. Baluch A. H. (2022). Synthesis and characterization of cobalt-doped ferrites for biomedical applications. ACS Omega 8, 3755–3761. 10.1021/acsomega.2c05226 PMC9893469 36743044

[B42] Kokare M. K. Jadhav N. A. Singh V. Rathod S. M. (2019). Effect of Sm^3+^ substitution on the structural and magnetic properties of Ni-Co nanoferrites. Opt. Laser Technol. 112, 107–116. 10.1016/j.optlastec.2018.10.045

[B43] Kulkarni V. D. Rathod S. M. (2016). Structural, morphological and optical properties of La^3+^ doped Co-Zn nanoferrite. IJSR 5.

[B44] Kumari N. Kumar V. Singh S. K. (2014). Synthesis, structural and dielectric properties of Cr^3+^ substituted Fe_3_O_4_ nano-particles. Ceram. Int. 40, 12199–12205. 10.1016/j.ceramint.2014.04.061

[B45] Li S. Pan J. Gao F. Zeng D. Qin F. He C. (2021). Structure and magnetic properties of coprecipitated nickel-zinc ferrite-doped rare earth elements of Sc, Dy, and Gd. J. Mat. Sci. Mat. Electron. 32, 13511–13526. 10.1007/s10854-021-05928-0

[B46] Lumina M. M. Anand S. Vinosel V. M. Janifer M. A. Pauline S. Manikandan A. (2018). Effect of lattice strain on structure, morphology and magneto-dielectric properties of spinel NiGd_x_Fe_2-x_O_4_ ferrite nano-crystallites synthesized by sol-gel route. J. Magn. Magn. Mat. 466, 238–251. 10.1016/j.jmmm.2018.07.017

[B47] Mahmood A. Maqsood A. (2021). Physical properties, magnetic measurements, dielectric relaxation, and complex impedance studies of cobalt-doped zinc ferrite nanoparticles. Appl. Nanosci. 11, 2311–2336. 10.1007/s13204-021-02007-y

[B48] Maksoud M. I. A. A. El-Ghandour A. El-Sayyad G. S. Fahim R. A. El-Hanbaly A. H. Bekhit M. (2020). Unveiling the effect of Zn^2+^ substitution in enrichment of structural, magnetic, and dielectric properties of cobalt ferrite. J. Inorg. Organomet. Polym. Mat. 30, 3709–3721. 10.1007/s10904-020-01523-8

[B49] Mariño-Castellanos P. Guerrero F. Romaguera-Barcelay Y. Goveia-Alcaide E. Cotta E. Leyet Y. (2021). Effect of La^3+^ cation solubility on the structural, magnetic and electrical properties of barium hexaferrite. Ceram. Int. 47, 8236–8247. 10.1016/j.ceramint.2020.11.183

[B50] Mısırlıoğlu B. S. Çakır Ö. Calik H. Cakir-Koc R. (2022). Assessment of structural and cytotoxic properties of cobalt ferrite nanoparticles for biomedical applications. Inorg. Nano-Metal Chem. 52, 57–62. 10.1080/24701556.2020.1862216

[B51] Mohamed M. B. Wahba A. M. (2014). Structural, magnetic, and elastic properties of nanocrystalline Al-substituted Mn_0.5_Zn_0.5_Fe_2_O_4_ ferrite. Ceram. Int. 40, 11773–11780. 10.1016/j.ceramint.2014.04.006

[B52] Mugutkar A. B. Gore S. K. Patange S. M. Mane R. S. Raut S. D. Shaikh S. F. (2022). Ammonia gas sensing and magnetic permeability of enhanced surface area and high porosity lanthanum substituted Co–Zn nano ferrites. Ceram. Int. 48, 15043–15055. 10.1016/j.ceramint.2022.02.033

[B53] Mugutkar A. B. Gore S. K. Tumberphale U. B. Jadhav V. V. Mane R. S. Patange S. M. (2020). The role of La^3+^ substitution in modification of the magnetic and dielectric properties of the nanocrystalline Co-Zn ferrites. J. Magn. Magn. Mat. 502, 166490. 10.1016/j.jmmm.2020.166490

[B54] Muskan A. Kumar N. Singh R. K. Kumar P. Siddique M. M. H. (2024). Rare earth (Nd^3+^) mediated structural, magnetic, ferroelectric properties of cobalt ferrite Nanomaterials for its varied applications. J. Indian Chem. Soc. 101, 101214. 10.1016/j.jics.2024.101214

[B55] Mustafa G. Khalid M. Chandio A. D. Shahzadi K. Uddin Z. Khan J. K. (2022). Dielectric, impedance, and modulus spectroscopic studies of lanthanum-doped nickel spinel ferrites NiLa_x_Fe_2-x_O_4_ nanoparticles. J. Sol-Gel Sci. Technol. 101, 596–605. 10.1007/s10971-020-05359-z

[B56] Nikam D. S. Jadhav S. V. Khot V. M. Ningthoujam R. S. Hong C. K. Mali S. S. (2014). Colloidal stability of polyethylene glycol functionalized Co_0.5_Zn_0.5_Fe_2_O_4_ nanoparticles: effect of pH, sample and salt concentration for hyperthermia application. RSC Adv. 4, 12662–12671. 10.1039/c3ra47319h

[B57] Nikumbh A. K. Pawar R. Nighot D. Gugale G. Sangale M. Khanvilkar M. (2014). Structural, electrical, magnetic and dielectric properties of rare-earth substituted cobalt ferrites nanoparticles synthesized by the co-precipitation method. J. Magn. Magn. Mat. 355, 201–209. 10.1016/j.jmmm.2013.11.052

[B58] Pandit R. Sharma K. K. Kaur P. Kumar R. (2014). Cation distribution controlled dielectric, electrical and magnetic behavior of In^3+^ substituted cobalt ferrites synthesized via solid-state reaction technique. Mat. Chem. Phys. 148, 988–999. 10.1016/j.matchemphys.2014.09.009

[B59] Patil B. B. Pawar A. D. Barate S. S. Ghodake J. S. Thorat J. B. Shinde T. J. (2023). Impact of La^3+^ substitution on electrical, magnetic, dielectric and optical properties of Ni_0.7_Cu_0.1_Zn_0.2_La_x_Fe_2–x_O_4_ (0 < x < 0.035) system. J. Rare Earth. 41, 740–746. 10.1016/j.jre.2022.03.023

[B60] Phor L. Kumar V. (2019). Structural, magnetic and dielectric properties of lanthanum substituted Mn_0.5_Zn_0.5_Fe_2_O_4_ . Ceram. Int. 45, 22972–22980. 10.1016/j.ceramint.2019.07.341

[B61] Reddy R. A. Rao K. R. Rajesh Babu B. Kumar G. K. Rajesh C. Chatterjee A. (2022). Structural, electrical and magnetic properties of cobalt ferrite with Nd^3+^ doping. Rare Met. 41, 240–245. 10.1007/s12598-019-01285-4

[B62] Ren X. Xu G. (2014). Electromagnetic and microwave absorbing properties of NiCoZn-ferrites doped with La^3+^ . J. Magn. Magn. Mat. 354, 44–48. 10.1016/j.jmmm.2013.10.056

[B63] Sanchez-Lievanos K. R. Stair J. L. Knowles K. E. (2021). Cation distribution in spinel ferrite nanocrystals: characterization, impact on their physical properties, and opportunities for synthetic control. Inorg. Chem. 60, 4291–4305. 10.1021/acs.inorgchem.1c00040 33734686

[B64] Sati P. C. Arora M. Chauhan S. Kumar M. Chhoker S. (2014). Effect of Dy substitution on structural, magnetic and optical properties of BiFeO_3_ ceramics. J. Phys. Chem. Solids. 75, 105–108. 10.1016/j.jpcs.2013.09.003

[B65] Shabbir S. Khalid B. Sehrish H. Iqbal M. T. Morley N. Anwar H. (2024). Exploring the structural, morphological, optical, and dielectric properties, along with photocatalytic performance of La-doped SrFeO_3_ nanofibers. Mat. Res. Bull. 179, 112970. 10.1016/j.materresbull.2024.112970

[B66] Shoba M. Kaleemulla S. (2017). Structural, optical and dielectric studies of Er substituted zinc ferrite nanospheres. J. Phys. Chem. Solids 111, 447–457. 10.1016/j.jpcs.2017.08.028

[B67] Sumalatha E. Kumar N. H. Edukondalu A. Ravinder D. (2022). Effect of La^3+^ ion doped Co-Zn nano ferrites: structural, optical, electrical and magnetic properties. Inorg. Chem. Commun. 146, 110200. 10.1016/j.inoche.2022.110200

[B68] Suo N. Sun A. Yu L. Zuo Z. Pan X. Zhang W. (2021). Effect of different rare earth (RE = Y^3+^, Sm^3+^, La^3+^, and Yb^3+^) ions doped on the magnetic properties of Ni–Cu–Co ferrite nanomagnetic materials. J. Mat. Sci. Mat. Electron. 32, 246–264. 10.1007/s10854-020-04762-0

[B69] Suo N. Sun A. Yu L. Zuo Z. Zhao X. Zhang W. (2020). Effect of Al^3+^ ion-substituted Ni–Mg–Co ferrite prepared by sol–gel auto-combustion on lattice structure and magnetic properties. Appl. Phys. A Mat. Sci. Process. 126, 183. 10.1007/s00339-020-3361-7

[B70] Tanbir K. Ghosh M. P. Singh R. K. Kar M. Mukherjee S. (2020). Effect of doping different rare earth ions on microstructural, optical, and magnetic properties of nickel–cobalt ferrite nanoparticles. J. Mat. Sci. Mat. Electron. 31, 435–443. 10.1007/s10854-019-02546-9

[B71] Thakur P. Sharma R. Kumar M. Katyal S. Barman P. Sharma V. (2019). Structural, morphological, magnetic and optical study of co-precipitated Nd^3+^ doped Mn-Zn ferrite nanoparticles. J. Magn. Magn. Mat. 479, 317–325. 10.1016/j.jmmm.2019.02.048

[B72] Xue Z. Hou Z. Ju J. Gao L. Zhang J. Peng Y. (2022). The cation distributions of Zn-doped normal spinel MgFe_2_O_4_ ferrite and its magnetic properties. Materials 15, 2422. 10.3390/ma15072422 35407754 PMC8999915

[B73] Zhang W. Sun A. Zhao X. Suo N. Yu L. Zuo Z. (2019). Structural and magnetic properties of La^3+^ ion doped Ni–Cu–Co nano ferrites prepared by sol–gel auto-combustion method. J. Sol-Gel Sci. Technol. 90, 599–610. 10.1007/s10971-019-04941-4

